# Increased homeostatic cytokines and stability of HIV-infected memory CD4 T-cells identify individuals with suboptimal CD4 T-cell recovery on-ART

**DOI:** 10.1371/journal.ppat.1009825

**Published:** 2021-08-27

**Authors:** Maria Pino, Susan Pereira Ribeiro, Amélie Pagliuzza, Khader Ghneim, Anum Khan, Emily Ryan, Justin L. Harper, Colin T. King, Sarah Welbourn, Luca Micci, Sol Aldrete, Keith A. Delman, Theron Stuart, Michael Lowe, Jason M. Brenchley, Cynthia A. Derdeyn, Kirk Easley, Rafick P. Sekaly, Nicolas Chomont, Mirko Paiardini, Vincent C. Marconi

**Affiliations:** 1 Division of Microbiology and Immunology, Yerkes National Primate Research Center, Emory University, Atlanta, Atlanta, Georgia, United States of America; 2 Department of Pathology and Laboratory Medicine, Emory School of Medicine, Emory University, Atlanta, Georgia, United States of America; 3 Centre de Recherche du CHUM and Department of Microbiology, Infectious Diseases and Immunology, Université de Montréal, QC, Canada; 4 Department of Biostatistics and Bioinformatics, Rollins School of Public Health, Emory University, Atlanta, Georgia, United States of America; 5 Emory Vaccine Center, Yerkes National Primate Research Center, Emory University, Atlanta, Georgia, United States of America; 6 Division of Infectious Diseases, Emory University School of Medicine, Atlanta, Georgia, United States of America; 7 Division of Surgical Oncology, Department of Surgery, Winship Cancer Institute, Emory University, Atlanta, Georgia, United States of America; 8 Emory Vaccine Center, Emory University, Hope Clinic, Decatur, Georgia, United States of America; 9 Barrier Immunity Section, Laboratory of Viral Diseases, National Institutes of Allergy and Infectious Diseases (NIAID), National Institutes of Health (NIH), Bethesda, Maryland, United States of America; 10 Atlanta Veterans Affairs Medical Center, Atlanta, Georgia, United States of America; University of Pennsylvania, UNITED STATES

## Abstract

Clinical outcomes are inferior for individuals with HIV having suboptimal CD4 T-cell recovery during antiretroviral therapy (ART). We investigated if the levels of infection and the response to homeostatic cytokines of CD4 T-cell subsets contributed to divergent CD4 T-cell recovery and HIV reservoir during ART by studying virologically-suppressed immunologic responders (IR, achieving a CD4 cell count >500 cells/μL on or before two years after ART initiation), and virologically-suppressed suboptimal responders (ISR, did not achieve a CD4 cell count >500 cells/μL in the first two years after ART initiation). Compared to IR, ISR demonstrated higher levels of HIV-DNA in naïve, central (CM), transitional (TM), and effector (EM) memory CD4 T-cells in blood, both pre- and on-ART, and specifically in CM CD4 T-cells in LN on-ART. Furthermore, ISR had higher pre-ART plasma levels of IL-7 and IL-15, cytokines regulating T-cell homeostasis. Notably, pre-ART PD-1 and TIGIT expression levels were higher in blood CM and TM CD4 T-cells for ISR; this was associated with a significantly lower fold-changes in HIV-DNA levels between pre- and on-ART time points exclusively on CM and TM T-cell subsets, but not naïve or EM T-cells. Finally, the frequency of CM CD4 T-cells expressing PD-1 or TIGIT pre-ART as well as plasma levels of IL-7 and IL-15 predicted HIV-DNA content on-ART. Our results establish the association between infection, T-cell homeostasis, and expression of PD-1 and TIGIT in long-lived CD4 T-cell subsets prior to ART with CD4 T-cell recovery and HIV persistence on-ART.

## Author summary

A major obstacle to curing HIV infection is our incomplete understanding of the mechanisms regulating the immunologic reconstitution of CD4 T-cells and the establishment and persistence of the latent HIV reservoir. In particular, we still do not know whether and to what extent the relative distribution of HIV infection within the various CD4 T-cell subsets influences: (i) the magnitude of the CD4 T-cell reconstitution, and (ii) the size of the persistent HIV reservoir after ART. In this study, we found that immunological suboptimal responders (ISR), people with HIV having a suboptimal CD4 T-cell recovery during ART, compared with immunological responders (IR) presented: i) higher levels of HIV-DNA in multiple CD4 T-cell subsets, and ii) higher expression of PD-1 and TIGIT exclusively on CM and TM CD4 T-cells pre-ART, indicating higher HIV reservoir maintenance. Paradoxically, we found increased plasma levels of IL-7 and IL-15 (T-cell homeostatic cytokines) be associated with suboptimal CD4 T-cell recovery during ART, which could be explained by the lack of response to these cytokines in CD4 T-cells from ISR. Altogether, these data identify a greater abundance of immune checkpoint molecules, an increased maintenance of HIV infection in long-lived CD4 T-cell subsets, and a different responsiveness to IL-7 and IL-15 as key features of and a mechanism for suboptimal CD4 T-cell recovery in ART-treated, people with HIV.

## Introduction

For more than ten years, multiple studies have demonstrated the relationship between the immunologic response after antiretroviral therapy (ART) and survival for virologically suppressed individuals with HIV [1–11]. There is a decisively increased risk of both serious AIDS and non-AIDS conditions for individuals who have either poor CD4 T-cell recovery or persistent inflammation [12–19]. An entire section of the U.S. Department of Health and Human Services Guidelines for the Use of Antiretroviral Agents in Adults and Adolescents Living with HIV is dedicated to the management of this population of patients [20]. Existing strategies to enhance CD4 T-cell recovery or reduce immune activation for these individuals have been largely unsuccessful. Age, nadir CD4 cell count, CD4/CD8 ratio, baseline inflammation, along with many other factors, have been implicated to cause this condition [21–28]. We have previously shown that early post-infection CD4 T-cell counts are able to predict CD4 T-cell recovery independent of CD4 nadir [29]. This implies that events proximal to infection dictate downstream phenomena. In this study, we have classified the study cohort as virologically-suppressed immunologic responders (IR, achieving a CD4 cell count >500 cells/μL on or before two years after ART initiation), and virologically-suppressed suboptimal responders (ISR, did not achieve a CD4 cell count >500 cells/μL in the first two years after ART initiation). Immunologic features were confirmed with analyses performed in a subset of individuals of the same cohort using a more restrictive threshold of <350 CD4 cells/μL ≥2 years on-ART, referred to as immunologic non-responders (INR) [30,31].

Central Memory T-cells (CM) are long-lived cells that play a crucial role in the maintenance of CD4 T-cell homeostasis. Indeed, studies in Simian immunodeficiency virus (SIV)_mac_-infected rhesus macaques (RM; a non-natural host for SIV that progress to AIDS) showed that the levels of infection and depletion of CM cells dictate the *tempo* of progression to AIDS [32], and that vaccine-induced preservation of CD4 CM T-cells predicts survival from SIV challenge [33,34]. In stark contrast to pathogenic SIV infection in macaques, natural SIV infection of sooty mangabeys (SM) is typically nonpathogenic despite high viremia. We observed that a key feature of non-pathogenic SIV-infection of SM is the low level of virus infection of CM T-cells as compared to both CD4 effector memory (EM) T-cells of SM and CM CD4 T-cells of RM [35,36]. Since CM cells are a critical population trafficking to lymph node (LN) tissue, this compartment may play an important role in determining CD4 T-cell recovery and ongoing inflammation after ART initiation. EM T-cells, which are relatively short-lived cells and home to peripheral lymphoid tissues, are easily infected by HIV in humans. If CM T-cells are relatively spared during acute HIV infection, it is conceivable that the rapid turnover of EM T-cells would not interfere with T-cell homeostasis during chronic infection and could permit a more rapid recovery after ART. Naïve (N) T-cells have a longer half-life than memory cells (1–8 years) and have variable susceptibility to HIV infection [37].

In an analysis of two AIDS Clinical Trials Group (ACTG) studies, higher levels of HIV reservoir in bulk CD4 T-cells before ART initiation were associated with higher levels while on-ART, and a similar association was observed for markers of immune activation, proliferation and inflammation. Baseline reservoir measurements were also correlated with T-cell activation before ART, but this association did not persist after ART [38]. In contrast, Khoury and colleagues showed in a cross-sectional study of virologically-suppressed individuals (CD4 cell counts > 350) that levels of T-cell activation did correlate with reservoir measures in blood, LN and rectal tissue [39]. Both studies measured HIV-DNA content in bulk CD4 T-cells. However, the overall pool of CD4 T-cells is constituted by several subsets that significantly differ in term of activation, life-span, and susceptibility to HIV infection. What remains completely unknown is whether the relative distribution of infected T-cell subsets pre-ART and their maintenance during ART influences post-ART CD4 cell count and inflammation.

Furthermore, it has been hypothesized that expression of co-inhibitory receptors (Co-IR) can contribute to maintenance of latently-infected CD4 T-cells [40]. Consistent with this hypothesis, CD4 T-cells expressing programmed cell death protein-1 (PD-1), T-cell immunoglobulin and immunoreceptor tyrosine-based inhibitory motif domains (TIGIT), T-cell immunoglobulin and mucin domain-containing protein-3 (TIM-3) or cytotoxic T lymphocyte-associated antigen-4 (CTLA-4) have been shown to have the greatest contribution to the reservoir pool, including the replication competent virus, among ART-treated individuals and predict viral rebound after treatment interruption [41–44]. Based on these findings, we sought to address two important questions for HIV pathogenesis and persistence. First, we determined whether differential infection of and Co-IR expression in specific CD4 T-cell subsets in blood and LN during ART discriminate between immunologic suboptimal responders (ISR) and immunologic responders (IR). Second, we investigated whether infection of and Co-IR expression in specific CD4 T-cell subsets as well as levels of cytokines that promote T-cell homeostasis prior to ART initiation predicted either suboptimal CD4 T-cell recovery or persistence of the HIV reservoir after ART. In this study, we define the HIV reservoir as the frequency of CD4 T-cell subsets harboring total and integrated HIV-DNA; due to the limited cell number in some of those subsets, we were not able to further define which of those genomes were intact and replication competent.

## Results

### Clinical factors discriminating IR and ISR participants

To determine the primary drivers of immunologic response to ART, a cohort of 32 participants was assembled from a population of individuals receiving care at any of the Emory University CFAR-affiliated HIV clinics. Mean age at ART start was 50 years, 91% were male and 72% were African-American (**S1 Table**). Most of these patients were initiated on ART during an advanced stage of disease due to late diagnosis and presentation to healthcare. The mean year of ART initiation was 4.3 ± 4.1 years and 72% of patients received a protease inhibitor or non-nucleoside reverse transcriptase inhibitor-based ART regimen. The mean CD4 nadir was 182 ± 145 cells/μL and the mean CD4 T-cells at ART initiation (baseline) was 217 ± 179 cells/μL. The mean CD4/CD8 ratio, considered the gold standard to measure immune reconstitution [27,28,45], was 0.35 ± 0.31, and 0.83 ± 0.5 at pre-, and on-ART, respectively. The mean duration of ART at the date of the last available sample was 4 ± 2 years. Of the total cohort, 13 participants were categorized as IR (achieving a CD4 count >500 cells/μL on or before two years after ART initiation), and 19 were ISR (did not achieve a CD4 cell count >500 cells/μL in the first two years after ART initiation). The characteristics that differed significantly between IR and ISR were the mean CD4 nadir (321 ± 109 cells/μL versus 86 ± 67 cells/μL, respectively), mean baseline CD4 cell count before ART initiation (390 cells/μL versus 99 cells/μL, respectively), pre-ART CD4/CD8 ratio (0.58 versus 0.18, respectively), and on-ART CD4/CD8 ratio (1.1 versus 0.62, respectively). Of note, there were no significant differences in ART duration, time from HIV diagnosis to ART initiation, and ART regimen. Pre-ART samples were available for 19 individuals (IR: n = 10; ISR: n = 9) and 32 individuals had samples available while on-ART (IR: n = 13; ISR: n = 19). Our data coincides with previous literature showing that low CD4/CD8 ratio predicts viremia, poor vaccine response and morbidity and mortality [26], and low CD4 nadir predicts poor immune reconstitution [46].

### CD4 recovery is associated with pre-ART CD4 counts and maintenance of long-lived subsets

**Fig 1:**
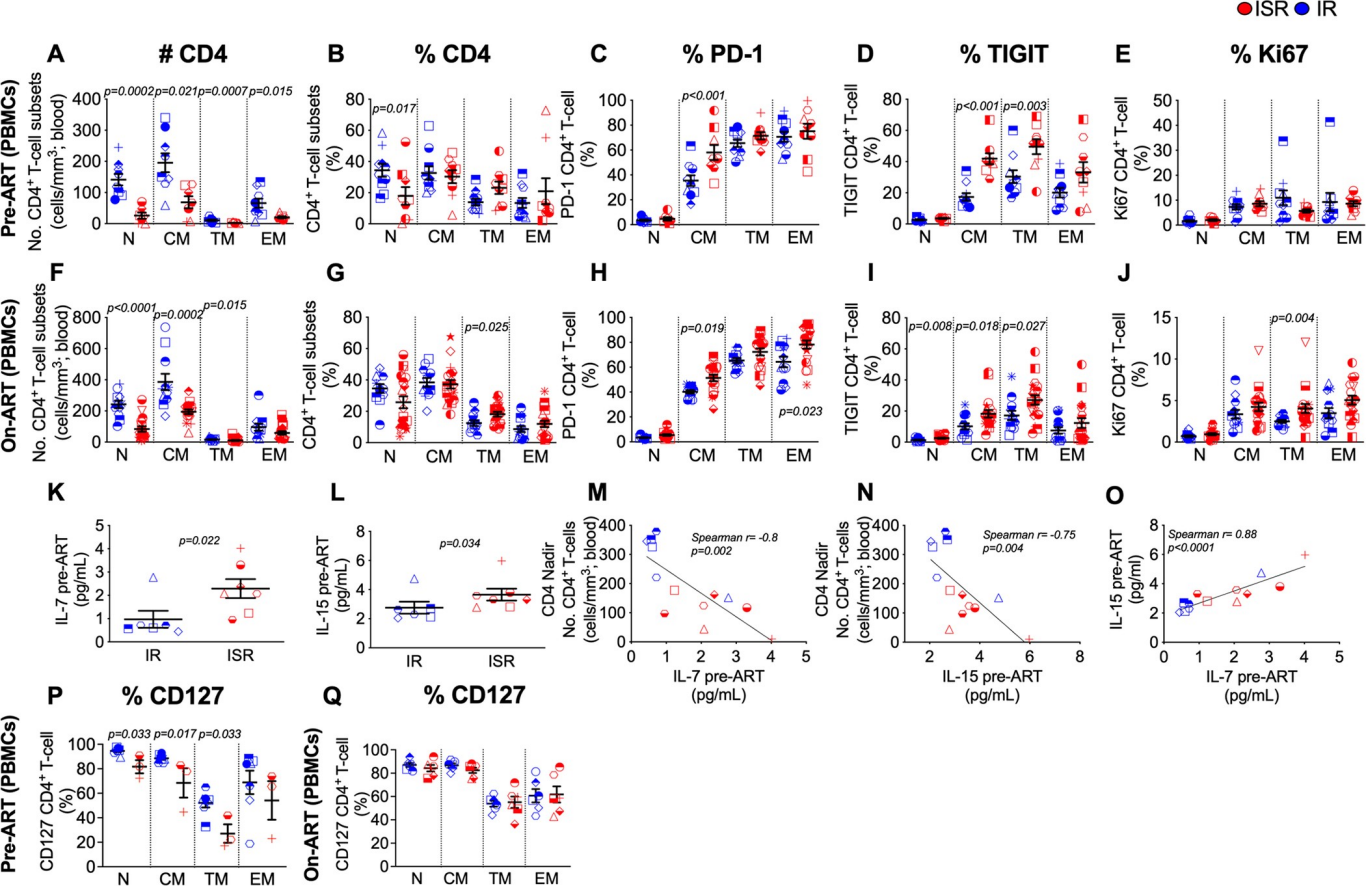
Suboptimal CD4 T-cell recovery is associated with low pre-ART CD4 cell counts, increased T-cell exhaustion of long-lived CD4 T-cell subsets and increased homeostatic cytokines. (A) Absolute counts (cells/mm^3^), and (B) frequency of CD4 T-cell subsets of PBMCs before ART initiation in immunologic responder (IR; blue, n = 10) and suboptimal responder (ISR; red, n = 9). (C) Expression of T-cell exhaustion markers PD-1, and (D) TIGIT on CD4 T-cell subsets of PBMCs before ART initiation (IR, n = 10; ISR, n = 9). (E) Expression of immune homeostatic marker, Ki67, on CD4 T-cell subsets of PBMCs before ART initiation (IR, n = 10; ISR, n = 9). (F) Absolute counts, and (G) frequency of CD4 T-cell subsets of PBMCs after ART initiation (IR, n = 12; ISR, n = 19). (H) Expression of PD-1, and (I) TIGIT on CD4 T-cell subsets of PBMCs after ART initiation (IR, n = 12; ISR, n = 19). (J) Expression of Ki67 on CD4 T-cell subsets of PBMCs after ART initiation (IR, n = 12; ISR, n = 19). (K) Levels of IL-7, and (L) IL-15 in plasma before ART initiation in IR and ISR (IR, n = 6; ISR, n = 7). (M) Correlations of IL-7, and (N) IL-15 levels in plasma before ART initiation with CD4 nadir. (O) Correlation of IL-17 with IL15 levels in plasma before ART initiation (IR, n = 6; ISR, n = 7). (P) Expression of CD127 on CD4 T-cell subsets of PBMCs before ART initiation (IR, n = 7; ISR, n = 3), and (Q) after ART initiation (IR, n = 6; ISR, n = 6). CD4 T-cell subsets included naïve (N), central memory (CM), transitional memory (TM), and effector memory (EM) cells. Averaged data are presented as the mean ± SEM. Repeated-measures analyses were performed with a means model (SAS MIXED Procedure, version 9.4) to generate statistical outcomes between IR and ISR in (A-J). Mann Whitney u-test was used in (K, L, P and Q). Spearman rank correlation test was used in (M, N, and O).

Higher pre-ART total CD4 cell count [47] and preservation of the naïve subset [48] have been associated with better CD4 cell recovery in clinical trials. In this population, absolute T-cell counts in blood for naïve (N; CD45RA+CD27+CCR7+), CM (CD45RA-CD27+CCR7+), transitional memory (TM; CD45RA-CD27-CCR7+), and EM (CD45RA-CD27-CCR7-) CD4 T-cells were greater before ART for IR compared to ISR (**Fig 1A**). During ART, differences between IR and ISR in blood CD4 T-cell counts were no longer significant for EM (**Fig 1F**). A representative gating strategy for the different CD4 T-cell subsets is shown in **S1 Fig**. When quantified as a relative frequency of total CD4 T-cells, only the naïve T-cell subset was significantly higher for IR compared to ISR pre-ART (**Fig 1B**), while the TM T-cell was significantly higher for ISR on-ART (**Fig 1G**). For CD8 T-cells, the differences were specific for the EM T-cell subset, in which percentage was significantly higher for ISR than IR pre-ART (**S2A Fig**) and on-ART (**S2B Fig**). The relative frequency of the different CD4 and CD8 T-cell subsets in LN on-ART was overall very similar between IR and ISR (**S2C and S2D Fig**).

### T-cell exhaustion and higher homeostatic cytokines are associated with suboptimal CD4 recovery

Co-IRs, such as PD-1 and TIGIT, are both up-regulated in CD4 and CD8 T-cells and trigger T-cell exhaustion during chronic HIV infection [49,50]. PD-1 expression on CD4 T-cells on-ART is associated with suboptimal CD4 T-cell recovery [51,52]; furthermore, the expression of PD-1, and additional Co-IRs [41] identify CD4 T-cells on-ART with a higher frequency of integrated HIV DNA [53]. Based on these data, we sought to determine in our cohort the frequency of CD4 and CD8 T-cells expressing PD-1, TIGIT, lymphocyte-activation gene 3 (LAG-3), or TIM-3. A composite analysis of all possible combinations (i.e., cells expressing any, 1, 2, 3, or all 4 Co-IRs) showed that the frequency of blood CD4 T-cells expressing one or more Co-IRs was higher in ISR pre-ART (**S3A Fig**) and on-ART (**S3B Fig**) as compared to IR. Similarly, ISR have higher frequency of LN CD4 T-cells expressing multiple Co-IRs on-ART (**S3C Fig**). These analyses on total CD4 T-cells identified PD-1 and TIGIT as the two Co-IRs accounting for the main differences between IR and ISR in blood and LN (**S4A, S4B, S4E,** and **S4F Fig**), while no significant differences were found in LAG-3 or TIM-3, neither pre- (blood) nor on-ART (blood and LN) (**S4C, S4D, S4G,** and **S4H Fig**). Based on these data, we next determined the expression of PD-1 or TIGIT for the different T-cell subsets. For blood CD4 T-cells at pre-ART, the frequency of PD-1+ cells was significantly higher for ISR exclusively on the CM T-cell subset (**Fig 1C**), whereas on-ART, ISR displayed a significantly higher frequency of CM and EM CD4 T-cells expressing PD-1 (**Fig 1H**). The increased levels of PD-1 expression on CM cells at pre-ART was specific for CD4 T-cells, since the frequency of CD8 T-cells expressing PD-1 at pre-ART was higher for ISR in the TM and EM T-cell but not the CM T-cell subset (**S5A Fig**). No differences of PD-1 expression were found for any of the CD8 T-cell subsets on-ART between IR and ISR (**S5D Fig**). The frequency of TIGIT expression was higher for ISR in blood CM and TM CD4+ T-cells at pre-ART (**Fig 1D**), and in naive, CM and TM CD4 T-cells on-ART (**Fig 1I**). No differences were observed for CD8 T-cells expressing TIGIT at pre-ART or on-ART (**S5B and S5E Fig**). Interestingly, the same immunologic features were confirmed with analyses performed in a subset of individuals of the same cohort using a more restrictive threshold of <350 CD4 cells/μL ≥2 years on-ART, referred to as immunologic non-responders (INR) [30,31]. Indeed, INR also demonstrated significantly higher PD-1 and TIGIT expression on CM T-cell subset when compared with IR at pre-ART (**S6 Fig**). Notably, the frequency of CD4 (**Fig 1E**) or CD8 T-cells (**S5C Fig**) expressing Ki67 pre-ART did not differ between IR and ISR across all subsets. During ART, the frequency of CD4+Ki67+ CM T-cells was also comparable between IR and ISR, with cycling levels being higher in ISR for the CD4 TM T-cell subset only (**Fig 1J**). No differences were observed for CD8+Ki67+ T-cells during ART (**S5F Fig**). The on-ART phenotype described for peripheral blood mononuclear cells (PBMCs) was confirmed in LN T-cells, with ISR showing a significantly higher frequency of naïve, CM and EM CD4 T-cells expressing PD-1 as well as CM, TM and EM CD4 T-cells expressing TIGIT as compared to IR (**S7A and S7B Fig**), while maintaining comparable levels of Ki67 expression (**S7C Fig**). The higher frequency of LN memory subsets expressing PD-1 and TIGIT was specific for CD4 T-cells, without any significant difference for the CD8 T-cell subsets (**S7D and S7E Fig**). Interestingly, when monitoring the expression of over 23 plasma cytokines, ISR had higher levels of IL-7 and IL-15, which promote T-cell survival, maintenance, and differentiation [54], than IR at pre-ART (**Fig 1K** and **1L**). No other cytokines were found to differ significantly between groups (**S2 Table**). Plasma levels of IL-7 and IL-15 pre-ART inversely correlated with CD4 nadir (**Fig 1M** and **1N**) and directly correlated with each other (**Fig 1O**). Although limited to a small subset of pre-ART samples for which cells were available (IR: n = 8, ISR = 3), we observed a statistically lower expression of α chain of the IL-7 receptor (CD127) for naïve, CM and TM CD4 T-cells from ISRs **(Fig 1P)**. However, our analysis showed no difference in CD127 expression in any of the CD4 T-cell subsets between ISRs and IRs on-ART **(Fig 1Q)**.

### Total and integrated HIV-DNA cellular levels are higher in ISR at baseline and during follow-up

**Fig 2:**
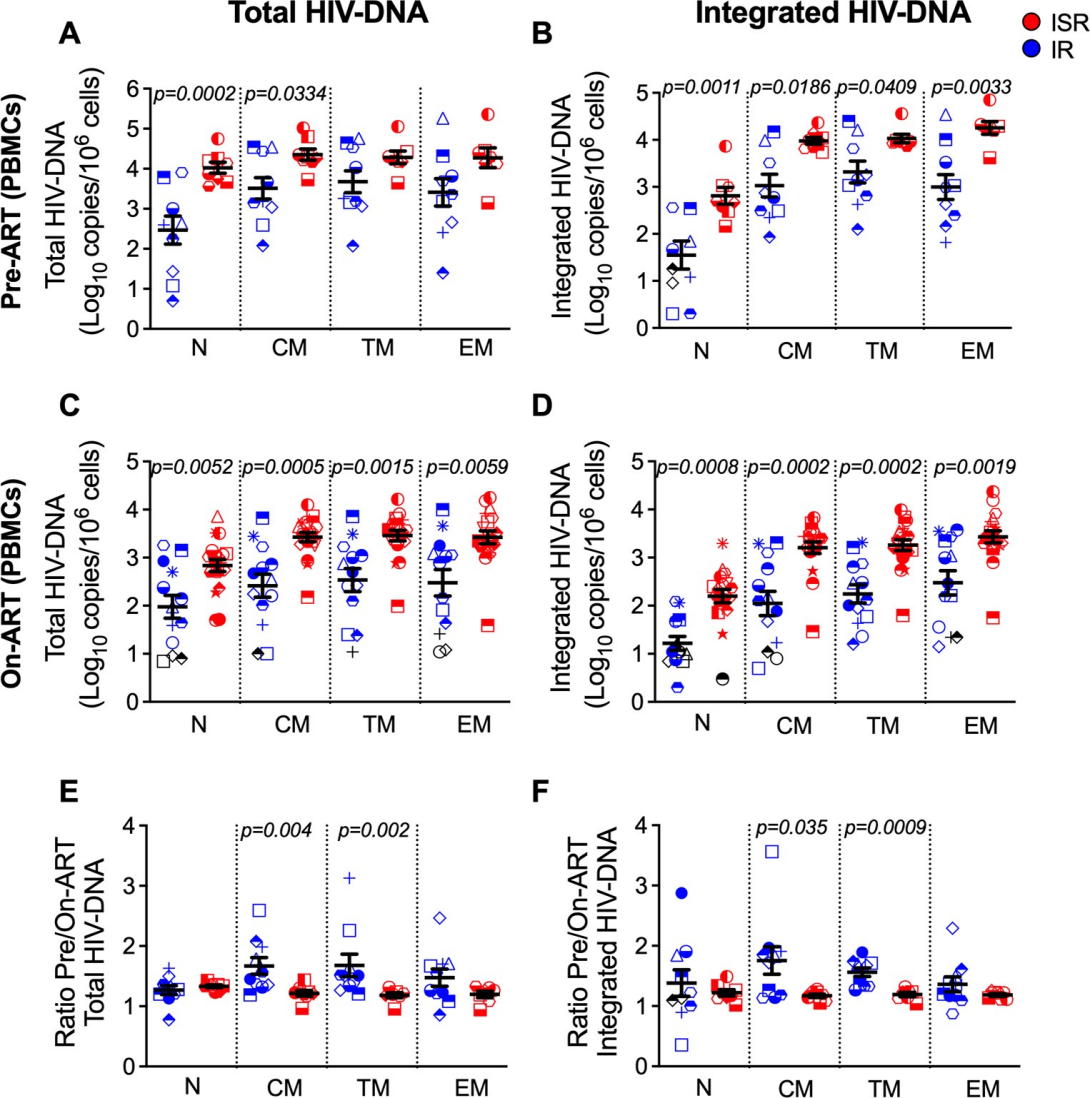
HIV-DNA reservoir levels are higher in ISR at baseline and during follow-up. (A) Total HIV-DNA cellular infection in peripheral blood CD4 T-cell subsets before ART initiation among immunologic responder (IR; blue, n = 10) and suboptimal responder (ISR; red, n = 8). (B) Integrated HIV-DNA cellular infection in peripheral blood CD4 T-cell subsets before ART initiation (IR, n = 10; ISR, n = 8). (C) Total HIV-DNA cellular infection in peripheral blood CD4 T-cell subsets after ART initiation (IR, n = 13; ISR, n = 19). (D) Integrated HIV-DNA cellular infection in peripheral blood CD4 T-cell subsets after ART initiation (IR, n = 13; ISR, n = 19). (E) Ratio pre-ART/On-ART ((Log_10_ copies pre-ART/10^6^ cells)/ (Log_10_ copies on-ART/10^6^ cells)) of total HIV-DNA, and (F) integrated HIV-DNA on blood CD4 T-cell subsets in immunologic responder (IR; blue, n = 10) and suboptimal responder (ISR; red, n = 8). CD4 T-cell subsets included naïve (N), central memory (CM), transitional memory (TM), and effector memory (EM) cells. Samples for which no target was detected are plotted at the limit of detection of the assay (calculated from cell input) and are represented as black symbols, and samples for which less than 3,000 cells were assayed were excluded from the analysis. Data show raw mean values and SEM. Repeated-measures analyses were performed with a means model (SAS MIXED Procedure, version 9.4) to generate statistical outcomes between IR and ISR.

Our findings showed that before ART initiation, ISR have a greater expression of PD-1 and TIGIT, markers previously associated with HIV persistence [41,50], on CM and TM CD4 T-cells, as well as higher plasma levels of IL-7 and IL-15. Based on these data, it is conceivable that, once infected, CM and TM CD4 T-cells could persist longer in ISR as they are maintained by IL-7 and IL-15 triggered survival and homeostatic proliferation signal pathways [55,56]. To this aim, we compared the relative levels of total and integrated HIV-DNA among different CD4 T-cell subsets between IRs and ISRs before and during ART. Briefly, CD4 T-cell subsets were sorted (**S1 Fig**), and then, total and integrated HIV-DNA were measured and represented as HIV reservoir normalized by 1*10^6^ cells to eliminate the variability in the amount of blood and sorted cells obtained from each participant. Before ART initiation, the levels of total HIV-DNA were significantly greater for ISR compared to IR in naïve and CM CD4 T-cells (**Fig 2A**) and the levels of integrated HIV-DNA were significantly greater for ISR for all blood CD4 T-cell subsets (**Fig 2B**). On-ART, both total and integrated HIV-DNA levels remained significantly larger for ISR than IR in all CD4 T-cell subsets (**Fig 2C and 2D**). When correcting the HIV reservoir by T-cell subset absolute counts, the reservoir size in CM T-cell subset remained significantly higher in ISR compared with IRs for both total and integrated HIV-DNA on-ART; however, EM T-cells in ISRs were also significantly higher for total HIV-DNA at pre-ART, and both total and integrated HIV-DNA on-ART (**S8 Fig**). Corrected HIV reservoir values by cell counts do not account for technical/biological variability in the amount of blood obtained or the number of cells present in each participant; as such, we considered representing HIV content data as relative frequency of HIV infection (log_10_/1*10^6^ cells) more accurate to determine the importance of HIV infection in different T-cell subsets between IR/ISRs.

Raw log_10_ means and estimated log_10_ means calculated with a univariate-repeated-measures model are shown in **Tables 1** and **S3**, respectively. As for the expression of PD-1 and TIGIT, increased levels of total and integrated HIV-DNA on-ART were confirmed in all CD4 T-cell subsets when analyses were performed only in the INR (<350 CD4 cells/μL ≥2 years on-ART) included in our cohort, as compared with IR (**S9 Fig**). Finally, total and integrated HIV-DNA levels on-ART were significantly higher for ISR than IR only for CM CD4 T-cells in LN (**S7G and S7H Fig**). Collectively, our findings indicate that ISR are characterized by higher frequency of cells harboring HIV-DNA both before and during ART, including in LN, and particularly so for the long-lived CM and TM T-cell subset.

**Table 1:** Estimated means and 95% confidence intervals for HIV-DNA levels in blood (PBMC) CD4 T-cell subsets (Naïve, CM, TM and EM cells) in univariate repeated-measures model.

CD4 T-cell subset	Pre-ART				On-ART		
		N	Log_10_ Mean ± SEM	P-value	N	Log_10_ Mean ± SEM	P-value
Naïve (total)	IR	10	2.481±0.209	0.0002	13	1.976±0.205	0.0052
	ISR	8	3.852±0.185		19	2.836±0.169	
Naïve (integrated)	IR	10	1.617±0.198	0.0011	13	1.219±0.182	0.0008
	ISR	8	2.750±0.207		18	2.199±0.155	
CM (total)	IR	10	3.609±0.188	0.0334	13	2.418±0.178	0.0005
	ISR	8	4.217± 0.182		19	3.427±0.147	
CM (integrated)	IR	10	3.090± 0.200	0.0186	13	2.050±0.189	0.0002
	ISR	8	3.825± 0.196		19	3.205±0.156	
TM (total)	IR	10	3.776± 0.196	0.20	13	2.533±0.184	0.0015
	ISR	7	4.153± 0.202		19	3.459±0.152	
TM (integrated)	IR	10	3.378± 0.170	0.0409	13	2.246±0.162	0.0002
	ISR	7	3.910± 0.167		19	3.260±0.134	
EM (total)	IR	10	3.422± 0.243	0.0510	13	2.477± 0.227	0.0059
	ISR	7	4.164± 0.252		19	3.423± 0.188	
EM (integrated)	IR	10	3.094± 0.207	0.0033	13	2.474± 0.196	0.0019
	ISR	7	4.120± 0.209		19	3.431± 0.162	

### ISR experienced a more limited pre- versus on-ART reduction in the frequency of HIV-DNA+ CM and TM CD4 T-cells

In addition to the frequency of infected cells at ART initiation, a key mechanism contributing to the long-term maintenance of HIV is the relative stability of the reservoir during ART. Taking advantage of our pre- and on-ART measures in the same cohort, we determined the magnitude of the reduction in HIV-DNA levels in CD4 T-cell subsets between pre-ART and on-ART (for example: log_10_ (10,000)/10^6^ cells pre-ART divided by log_10_ (100) copies/10^6^ cells on-ART is equivalent to 4 divided by 2, a 2-log change). While nearly all participants experienced a decline in total and integrated HIV-DNA levels in all CD4 T-cell subsets after initiating ART, the fold reduction was significantly larger for IR compared to ISR in the CM and TM T-cell subsets both for total (**Fig 2E**) and integrated (**Fig 2F**) HIV-DNA. Specifically, and despite the fact that the duration of ART was longer for ISR, the integrated HIV-DNA content decrease was larger for IR than for ISR (IR: 1.75-log fold reduction for CM and 1.56-log fold reduction for TM; ISR: 1.17-log fold reduction for CM and 1.21-log fold reduction for TM) (**Fig 2F**). Notably, in ISR the more limited reduction of the HIV-DNA content between pre- and on-ART was statistically significant only for CM and TM CD4 T-cells. Indeed, the decline was relatively similar between ISR and IR for total (**Fig 2E**) and integrated (**Fig 2F**) HIV-DNA in EM cells, and ISR had a higher reduction than IR in total and integrated HIV-DNA levels for naïve cells (**S10 Fig**; express as percentage reduction from pre-ART). To further highlight the heightened maintenance of CM and TM CD4 T-cells harboring HIV-DNA in ISR as compared to IR, we used a univariate repeated-measured model to estimate the mean slope/decline per year on-ART of the HIV-DNA content among the different CD4 T-cell subsets. As quantified in **Table 2**, the mean decline per year of total and integrated HIV-DNA content was significantly higher for IR as compared to ISR in TM cells (p = 0.04 for total, and p = 0.03 for integrated HIV-DNA) and a higher trend in CM cells (p = 0.07, and p = 0.08); but not for EM cells (p = 0.29, and p = 0.67). The mean decline per year was greater for ISR compared with IR for both total and integrated HIV-DNA in naïve cells. Based on the content of integrated HIV-DNA before ART initiation and its mean decline per year of ART, it is estimated that it will require 4-times longer for the HIV-DNA content in CM and TM CD4 T-cells to reach zero in ISR as compared to IR (**Table 2**). Collectively, our findings indicate that CM and TM CD4 T-cells of ISR are infected at a higher frequency before ART initiation, express higher levels of the Co-IRs PD-1 and TIGIT, and remain more stable during ART. These three mechanisms synergistically favor long-term maintenance of HIV-DNA in critical cellular reservoirs for ISR as compared to IR.

**Table 2:** Mean total and integrated HIV-DNA decay in CM, TM, EM, and naïve T-cells (log transformed) slope:decline/year and SE.

**Total HIV DNA in Naïve (log**_**10**_ **)**							
	All Patients		Immunologic Responders		Immunologic Suboptimal Responders		
	N	Total HIV DNA	N	Total HIV DNA	N	Total HIV DNA	P value
Mean Intercept (SE)	32	3.1652 (0.1893)	13	2.4624 (0.2351)	19	3.7156 (0.2220)	< .01
Mean slope:decline/year (SE)	32	-0.1552 (0.0295)	13	-0.1268 (0.0419)	19	-0.1843 (0.0366)	0.32
**Total HIV DNA in CM (log**_**10**_ **)**							
	All Patients		Immunologic Responders		Immunologic Suboptimal Responders		
	N	Total HIV DNA	N	Total HIV DNA	N	Total HIV DNA	P value
Mean Intercept (SE)	32	3.6999 (0.1789)	13	3.3932 (0.2145)	19	3.9636 (0.2134)	0.08
Mean slope:decline/year (SE)	32	-0.1346 (0.0384)	13	-0.2321 (0.0538)	19	-0.0986 (0.0432)	0.07
**Total HIV DNA in TM (log**_**10**_ **)**							
	All Patients		Immunologic Responders		Immunologic Suboptimal Responders		
	N	Total HIV DNA	N	Total HIV DNA	N	Total HIV DNA	P value
Mean Intercept (SE)	32	3.7060 (0.1759)	13	3.5462 (0.2169)	19	3.8981 (0.2228)	0.28
Mean slope:decline/year (SE)	32	-0.1190 (0.0397)	13	-0.2401 (0.0554)	19	-0.0804 (0.0453)	0.04
**Total HIV DNA in EM (log**_**10**_ **)**							
	All Patients		Immunologic Responders		Immunologic Suboptimal Responders		
	N	Total HIV DNA	N	Total HIV DNA	N	Total HIV DNA	P value
Mean Intercept (SE)	32	3.6171 (0.2003)	13	3.2633 (0.2484)	19	3.9299 (0.2539)	0.08
Mean slope:decline/year (SE)	32	-0.1164 (0.0419)	13	-0.1853 (0.0614)	19	-0.0980 (0.0508)	0.29
**Integrated HIV DNA in Naïve (log**_**10**_ **)**							
	All Patients		Immunologic Responders		Immunologic Suboptimal Responders		
	N	Integrated HIV DNA	N	Integrated HIV DNA	N	Integrated HIV DNA	P value
Mean Intercept (SE)	31	2.0602 (0.1752)	13	1.4934 (0.1959)	18	2.5309 (0.1980)	< .01
Mean slope:decline/year (SE)	31	-0.0476 (0.0344)	13	-0.0543 (0.0504)	18	-0.0553 (0.0401)	0.99
**Integrated HIV DNA in CM (log**_**10**_ **)**							
	All Patients		Immunologic Responders		Immunologic Suboptimal Responders		
	N	Integrated HIV DNA	N	Integrated HIV DNA	N	Integrated HIV DNA	P value
Mean Intercept (SE)	32	3.3703 (0.1865)	13	2.9471 (0.2183)	19	3.6862 (0.2147)	0.03
Mean slope:decline/year (SE)	32	-0.1331 (0.0369)	13	-0.2187 (0.0502)	19	-0.0959 (0.0414)	0.08
**Integrated HIV DNA in TM (log**_**10**_ **)**							
	All Patients		Immunologic Responders		Immunologic Suboptimal Responders		
	N	Integrated HIV DNA	N	Integrated HIV DNA	N	Integrated HIV DNA	P value
Mean Intercept (SE)	32	3.4368 (0.1676)	13	3.1820 (0.1955)	19	3.6767 (0.1998)	0.10
Mean slope:decline/year (SE)	32	-0.1144 (0.0362)	13	-0.2223 (0.0483)	19	-0.0771 (0.0399)	0.03
**Integrated HIV DNA in EM (log**_**10**_ **)**							
	All Patients		Immunologic Responders		Immunologic Suboptimal Responders		
	N	Integrated HIV DNA	N	Integrated HIV DNA	N	Integrated HIV DNA	P value
Mean Intercept (SE)	32	3.5895 (0.1815)	13	3.0302 (0.2197)	19	3.9736 (0.2171)	< .01
Mean slope:decline/year (SE)	32	-0.1230 (0.0309)	13	-0.1387 (0.0447)	19	-0.1128 (0.0392)	0.67

### Expression of PD-1 and TIGIT on CM CD4 T-cells and levels of IL-7 and IL-15 correlate with HIV-DNA content pre-ART and during ART

**Fig 3:**
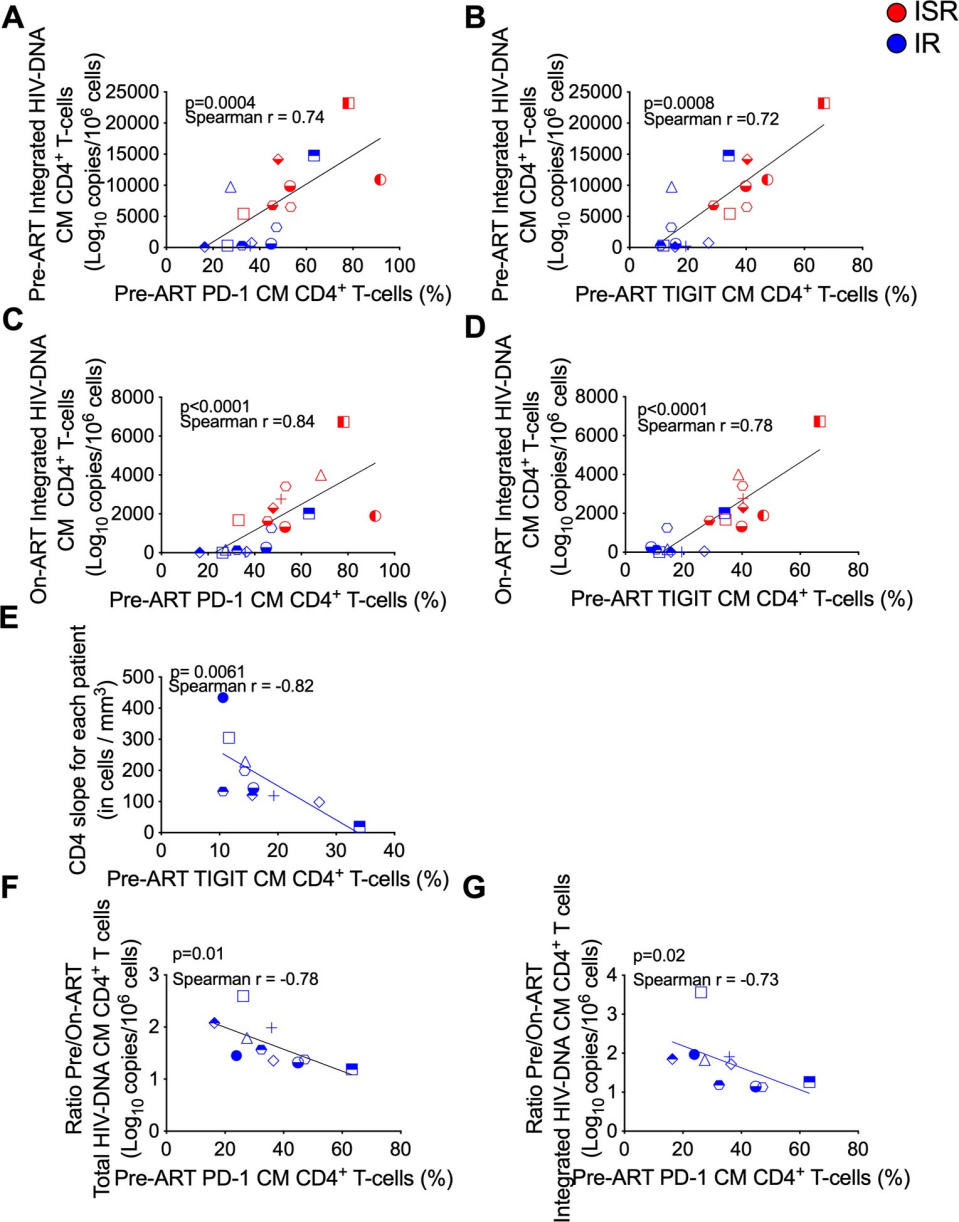
Expression of co-IRs on CD4 CM T-cells correlate with HIV-DNA content pre-ART and during ART. (A-D) Correlation of PD-1 (A, C), and TIGIT (B, D) expression levels on central memory (CM) cells at pre-ART with integrated HIV-DNA on CM cells at pre- (A, B; IR, blue; n = 10, ISR, red; n = 7), and at on-ART (C, D; IR n = 10; ISR n = 9) for all individuals with HIV (n = 18). (E) Correlation of TIGIT expression levels on CM at pre-ART with CD4 T-cell recovery (CD4 slope; cells/mm^3^) on immunologic responders (IR) (n = 10). (F) Correlation of PD-1 expression on CM cells at pre-ART with ratio pre-ART/On-ART ((Log_10_ copies pre-ART/10^6^ cells)/(Log_10_ copies on-ART/10^6^ cells)) of total, and (G) integrated HIV-DNA on CM cells on IR, individuals with HIV (n = 9). Spearman rank correlation test was used to determine correlations.

**Fig 4:**
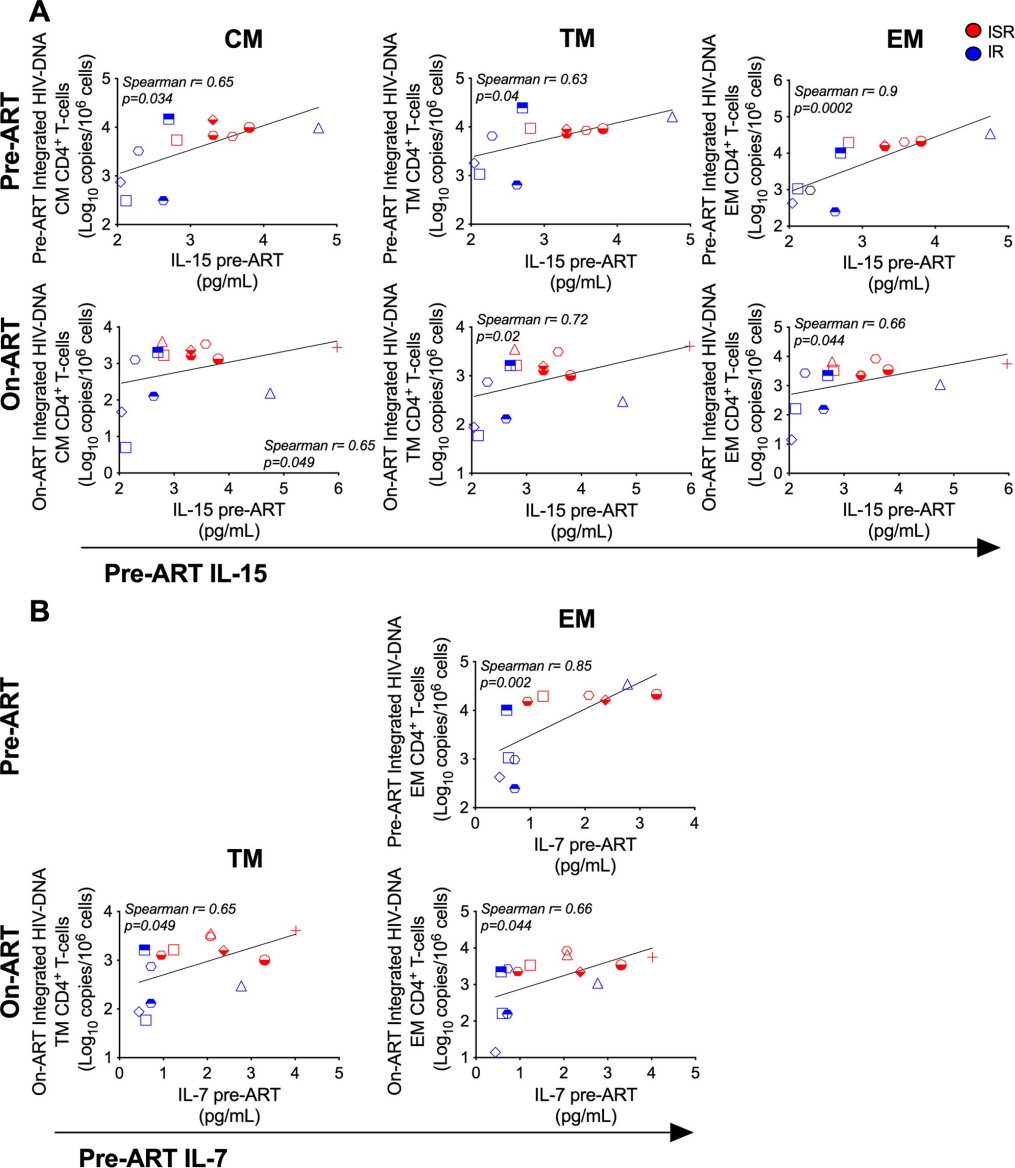
Pre-ART IL-15 and IL-7 plasma cytokine levels correlate with HIV reservoir in blood CM, TM, and EM subsets at pre-, and on-ART. (A) Correlation of IL-15 pre-ART cytokine plasma levels with integrated HIV-DNA in CD4 T-cell subsets at pre-ART (top panels, n = 11; IR n = 6, ISR n = 5), and on-ART (bottom panels, n = 13; IR n = 6, ISR n = 7). (B) Correlation of IL-7 pre-ART cytokine plasma levels with integrated HIV-DNA in CD4 T-cell subsets at pre-ART (top panels, n = 11; IR n = 6, ISR n = 5), and on-ART (bottom panels, n = 13; IR n = 6, ISR n = 7). Blue indicates IR, red indicates ISR. CD4 T-cell subsets included naïve (N), central memory (CM), transitional memory (TM), and effector memory (EM) cells. Spearman rank correlation test was used to determine correlations among all individuals.

CD4 T-cells expressing PD-1, TIGIT, and LAG-3 have been found to harbor higher levels of HIV-DNA on-ART [41,53]. The findings that ISR have a more limited pre- versus on-ART reduction in the frequency of HIV-infected CM and TM CD4 T-cells and increased expression of PD-1 and TIGIT on the same subsets prompted us to investigate potential correlations between expression of PD-1 or TIGIT and HIV-DNA content among all participants. Intriguingly, the frequency of CM CD4 T-cells expressing PD-1 or TIGIT at pre-ART were strongly correlated with the levels of integrated HIV-DNA content in CM CD4 T-cells at pre-ART (**Fig 3A and 3B**) as well as during ART (**Fig 3C and 3D**). Similar correlations were confirmed for total HIV-DNA. We also found a decline in the CD4 T-cell recovery (CD4 slope) of about 21 cells/mm^3^ per year of ART for a 10% increase in pre-ART PD-1 expression in CM CD4 T-cells (p = 0.06; **S11A Fig**), and similar results were found in pre-ART TIGIT expression in CM CD4 T-cells (p = 0.013; **S11B Fig**). When analyses were restricted to IRs (but not ISRs), increased TIGIT (**Fig 3E**) expression on CM CD4 T-cells pre-ART was significantly correlated with reduced CD4 T-cell recovery (CD4 slope) on-ART, and CM PD-1 CD4 T-cell levels pre-ART negatively correlated with fold reduction (ratio pre-ART/on-ART) of total and integrated HIV-DNA in CM T-cell subsets (**Fig 3F and 3G**). Thus, expression of PD-1 and TIGIT in CM CD4 T-cells pre-ART could predict recovery of CD4 T-cells and the frequency of infected cells on-ART. Moreover, pre-ART levels of IL-15 positively correlated with integrated HIV-DNA in CD4 CM, TM and EM T-cell subsets both pre- and on-ART for all individuals (**Fig 4A**). Likewise, pre-ART IL-7 levels positively correlated with integrated HIV-DNA in EM cells pre-ART and TM and EM T-cell subsets on-ART (**Fig 4B**). Finally, at pre-ART, plasma levels of IL-15 (r = 0.63, p = 0.02) and IL-7 (r = 0.66, p = 0.01) positively correlated with the frequency of CM CD4 T-cells expressing TIGIT. These findings are consistent with a critical role of PD-1, TIGIT, IL-7, and IL-15 expression in regulating reservoir size and CD4 T-cell recovery.

### Lower proliferative capacity in response to homeostatic cytokines characterize CD4 T-cells of ISRs

**Fig 5:**
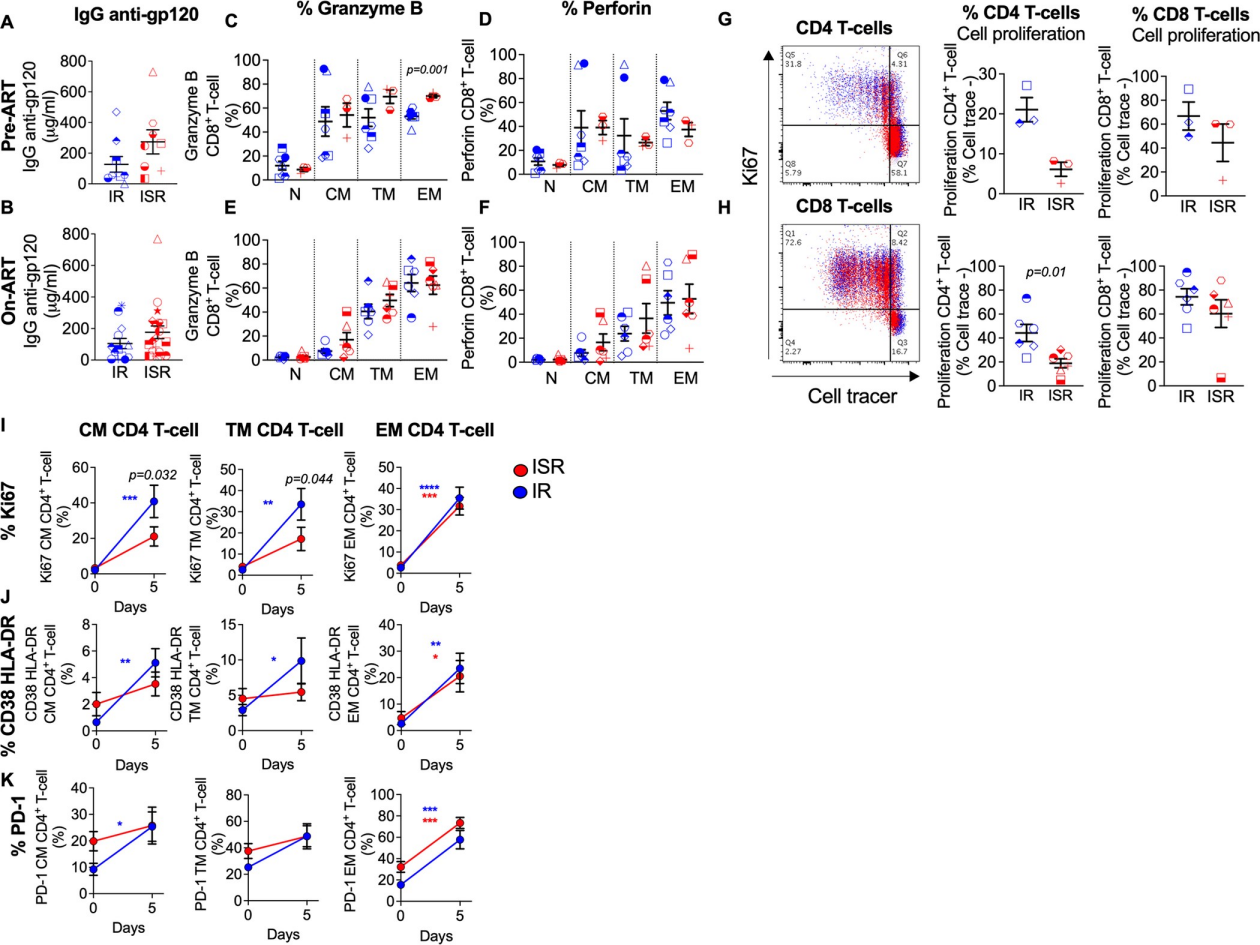
Lower response to homeostatic cytokines characterize CD4 T-cells of ISRs. Plasma IgG antibodies against HIV-gp120 (A) before ART (IR, n = 9; ISR, n = 8), (B) and after ART initiation (IR, n = 13; ISR, n = 19) in ISR and IR individuals. Expression of cytolytic markers, (C, E) granzyme B and (D, F) perforin on CD8 T-cell subsets of PBMCs before (IR, n = 7; ISR, n = 3), and after (IR, n = 6; ISR, n = 6) ART initiation. Cell proliferation capacity (cell trace -) of CD4 and CD8 T-cells at day 7 after IL-15+IL-7 stimulation of (G) pre- (IR, n = 3; ISR, n = 3), and (H) on-ART (IR, n = 6; ISR, n = 6) samples. (I) Expression of Ki67, (J) CD38 HLA-DR, and (K) PD-1 in sorted CD4 T-cell subsets (CM, TM, and EM T-cells) at baseline and day 5 after IL-15+IL-7 stimulation from on-ART ISR (n = 6) and IR (n = 6) individuals. Blue indicates IR, red indicates ISR. CD4 T-cell subsets included naïve (N), central memory (CM), transitional memory (TM), and effector memory (EM) cells. Averaged data are presented as the mean ± SEM. Mann Whitney u-test was used in (A-H). Two-way ANOVA with Sidak’s multiple comparisons test was used in (I, J, and K). Asterisks indicate comparisons between baseline and day 5 time point, and p-values indicate comparisons within IR and ISR at the same time point. * P<0.05, ** p<0.01, *** p<0.001, ****p≤ 0.0001.

To better understand the mechanisms contributing to the differences observed among IRs and ISRs in the pre- versus on-ART reduction of HIV-infected CM and TM CD4 T-cells (**Fig 2**), we first analyzed HIV-specific antibody responses and CD8 cytolytic T-cell responses. The concentration of plasma IgG antibodies against HIV-Envelope (**Fig 5A** and **5B**) and the expression of cytolytic markers, such as granzyme B and perforin, on different CD8 T-cell subsets (**Fig 5C–5F**) were overall similar in IRs and ISRs both pre- and on-ART (with the exception of EM CD8 T-cells in ISRs which express higher level of granzyme B than IRs pre-ART; **Fig 5C**). Since CD8 T-cell cytolytic responses and IgG antibodies were not inferior in ISRs compared to IRs, this mechanism would be unlikely to contribute to the reduced clearance rate of HIV-DNA observed among ISRs.

To reconcile the inefficient T-cell replenishment in blood (**Fig 1A**) for ISR despite higher pre-ART levels of IL-7 and IL-15 (**Fig 1K and 1L**), we compared the T-cell proliferative potential in response to homeostatic cytokines by Cell Trace dye dilution between ISR and IR in a small subset of individuals with samples available. The frequency of proliferating CD4 T-cells (cell trace low), but not of CD8 T-cells, was remarkably lower in ISRs than IRs. These results were observed both at pre-ART (IR: n = 3; ISR: n = 3) (**Fig 5G**) and on-ART (IR: n = 6; ISR: n = 6) (**Fig 5H**) time points and could explain, at least in part, why ISRs have a reduced ability to recover CD4 T-cell in blood (**Fig 1A**) despite higher levels of pre-ART IL-7 and IL-15 (**Fig 1K and 1L**). The reduced responsiveness to those cytokines was confirmed by assessing various cell markers in memory CD4 T-cell subsets sorted from on-ART PBMCs (IR: n = 6; ISR: n = 6). Upon *in vitro* stimulation with IL-7 and IL-15, Ki67 was significantly increased as compared to baseline on CM, TM, and EM CD4 T-cell subsets from IR (**Fig 5I**). Notably, in ISR the increased expression of Ki67 was significant as compared to baseline only for EM CD4 T-cells, with a variation identical to those measured in IRs. As a result, the frequency of CM or TM CD4+Ki67+ T-cells at day 5 post stimulation were significantly lower in ISRs than IRs (**Fig 5I**, p = 0.032 and p = 0.044, respectively). We observed a similar trend, significant increase from baseline in all CD4 T-cell subsets in IRs but only in EM CD4 T-cells for ISRs, for the expression of the activation markers CD38 and HLA-DR (**Fig 5J**), as well as PD-1 (**Fig 5K**). Altogether, these results suggest that, in response to IL-7 and IL-15 stimulation, CM and TM CD4 T-cell subsets from IR are more prone to activation, proliferation, and differentiation to effector T-cells as compared to the same cell subsets for ISR. We used the same experimental setting to determine whether IL-7+IL-15 stimulation differently impacts HIV-DNA and -RNA levels in IRs and ISRs, measured in the cell pellets or supernatant, respectively, collected from the sorted cell cultures explained above. HIV-RNA (**S12A Fig**) or HIV-DNA (**S12B Fig**) levels after 5 days of cytokine exposure did not significantly differ between IRs and ISRs.

Altogether, these data indicate that a lower proliferative capacity in response to homeostatic cytokines characterize CD4 T-cells of ISRs.

## Discussion

In this study, we found ISR, people with HIV having a suboptimal CD4 T-cell recovery during ART, to have a number of important immunologic and virologic distinctions from those individuals with robust CD4 T-cell recovery, both prior to and on-ART. Specifically, levels of total and integrated HIV-DNA in multiple CD4 T-cell subsets as well as plasma levels of IL-7 and IL-15 were significantly higher pre-ART for ISR. Most importantly, the reduction in total and integrated HIV-DNA levels between the pre- and on-ART time point was significantly lower exclusively in CM and TM CD4 T-cells of ISR than IR. Moreover, ISR showed higher expression of PD-1 and TIGIT exclusively on CM and TM CD4 T-cells pre-ART, with the frequency of CM CD4 T-cells expressing PD-1 or TIGIT pre-ART predicting the extent of CD4 T-cell recovery and HIV-DNA content during ART among all participants. Pre-ART levels of IL-15 positively correlated with integrated HIV-DNA in CD4 CM, TM and EM T-cell subsets both pre-ART and on-ART, and pre-ART levels of IL-7 positively correlated with integrated HIV-DNA in EM cells pre-ART and TM and EM T-cell subsets on-ART. Finally, we have identified a reduced proliferative response to homeostatic cytokines during *ex vitro* stimulation of CD4 T-cells from a small subset of ISRs that is specific for CM and TM subsets. Altogether, these data identify a greater abundance of immune checkpoint molecules, an increased maintenance of HIV infection in long-lived CD4 T-cell subsets, and a different responsiveness to IL-7 and IL-15 as key features of and a possible mechanism for suboptimal CD4 T-cell recovery in ART-treated, people with HIV.

The health, quality of life, and cost implications of this phenomenon are widespread. Persistent inflammation and suboptimal CD4 T-cell recovery despite virologic suppression has been implicated as causes of cognitive dysfunction [57], cardiovascular disease [6,16], pulmonary [58] and renal disease [59], diabetes [60], cancer [6,18,61], depression [62], and frailty [63] for individuals with HIV. Currently, effective strategies for improving CD4 T-cell recovery are lacking. Beyond a cure for HIV infection, this remains one of the most important issues for individuals receiving ART. Previous studies have shown a relationship between total HIV-DNA levels and CD4 T-cell recovery. For individuals already on treatment, HIV-DNA levels in PBMCs have been associated with reductions in the proportions of naïve CD4 and CD8 T-cells and concomitantly with an increase in the proportion of effector CD4 T-cells indicating that proviral DNA hinders immune recovery on-ART [64]. In a cross-sectional study, suboptimal CD4 T-cell recovery was associated with increased total HIV-DNA in both memory and naïve CD4 T-cells as well as increased IL-7 and thymic impairment [65]. Moreover, baseline levels of total central memory [66] as well as naïve T-cells [67] have been associated with CD4 T-cell recovery indicating the importance of preserving these subsets prior to ART initiation in order to permit maximal immunological response to treatment. Of note, in HIV infection, CM CD4 T-cells are key contributors to the pool of infected cells both *in vivo* and *in vitro* [68,69]. Consistent with the finding that these cells have a longer *in vivo* lifespan than resting EM T-cells, we showed that among ART-treated individuals with HIV, CM T-cells represent the largest reservoir of infected CD4 T-cells [53]. Therefore, individuals with a greater relative infection of the CM T-cell subset may have a longer persistence of the HIV reservoir which is confirmed in our study.

**Fig 6:**
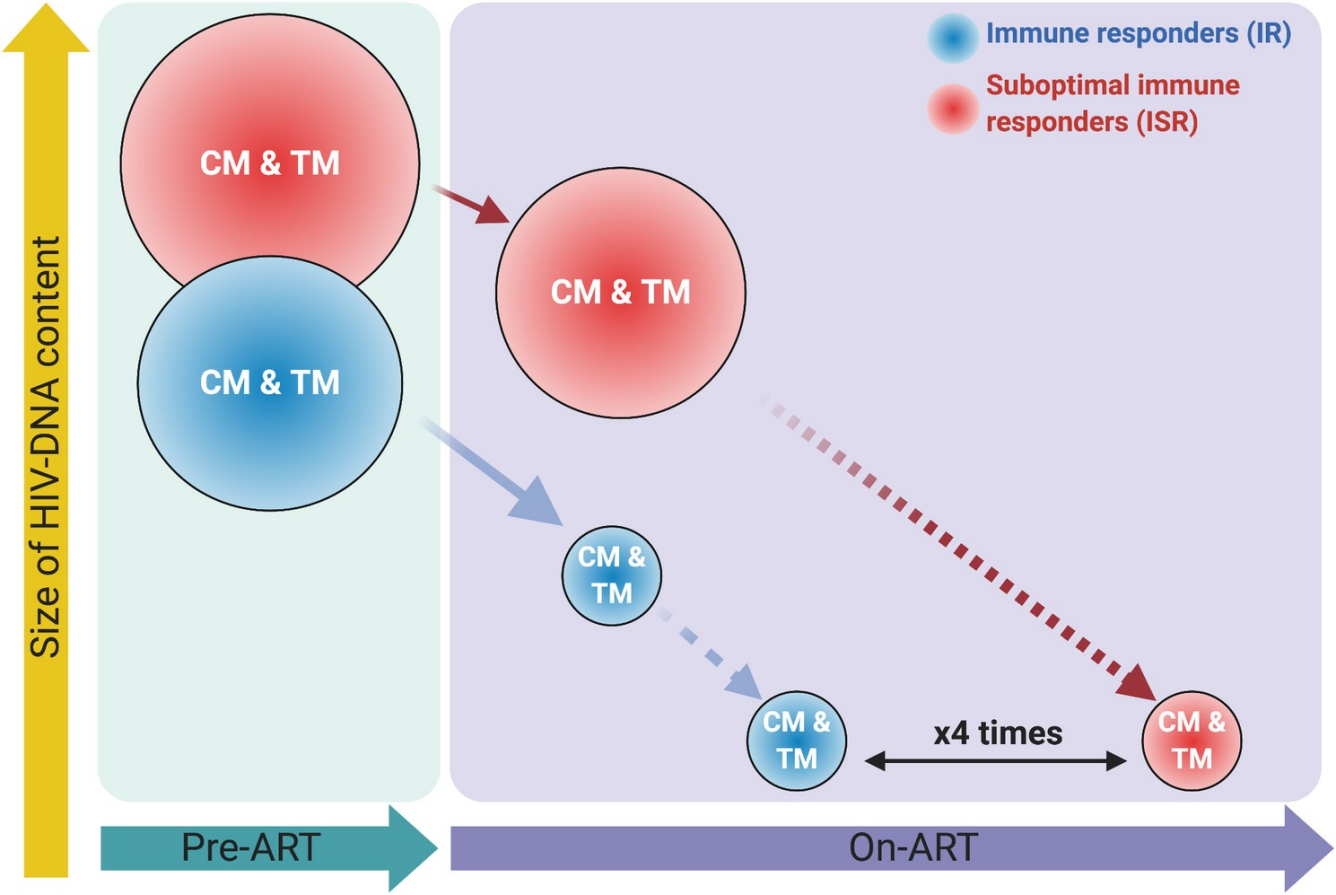
Schematic representation of latent reservoir dynamics after ART initiation over time in CM and TM CD4 T-cells from immunologic responders (IR; blue circles) and suboptimal immunologic responders (ISR; red circles). Size of the circles represents the level of co-IRs expression.

Our current study also showed that in ISR, the critical cellular reservoir of CM and TM CD4 T-cells [53] are (i) infected at higher frequency already before ART initiation and (ii) more stably maintained during ART. Combined, these two features highly differentiate the ability to clear the reservoir for ISR as compared to IR. For example, based on our data it can be estimated that to eradicate the HIV-DNA content in CM and TM CD4 T-cells during ART, it would require four times longer for ISR than IR (see **Fig 6**). Our estimates have been generated with only two experimental time points and using a stable fold-change, acknowledging that HIV-DNA levels decline faster at the beginning of ART and then much slower with ART continuation [70]. It is also important to note that variable reconstitution of uninfected and infected cells could overestimate and underestimate, respectively the actual decay of the original pre-ART HIV-DNA levels in different T-cell subsets. Furthermore, HIV reservoir correction by absolute counts would result in an inaccurate quantification due to variability in the amount of blood and number of purified cells obtained between different individuals; therefore, we followed the precedence of the vast majority of studies quantifying HIV reservoir which have represented these data in terms of relative frequency of HIV infection (log_10_/1*10^6^ of cells). Nevertheless, our estimates clearly highlight the increased contribution of CM and TM CD4 T-cells to the long-term maintenance of the HIV reservoir in ISR as compared to IR. Although the HIV-DNA reduction in EM CD4 T-cell subset was not significantly different between IRs and ISRs, this subset also contributes to the maintenance of the HIV reservoir during ART [71]. Recent studies have concluded that although the naïve CD4 T-cell subset has a lower frequency of HIV infection compared with memory CD4 T-cell subsets, these cells harbor more replication competent HIV-1 than previously thought, and that could be a significant contributor to HIV persistence [72,73] In our study, intact provirus could not be assessed due to the limited number of purified cells obtained. Collectively, these data indicate that an increased stability during ART for the HIV-infected, long-lived CM and TM cells discriminates ISR from IR, and contributes to the increased persistence of the viral reservoir in ISR.

As previously mentioned, we found increased plasma levels of IL-7 and IL-15 before initiation of ART to be associated with suboptimal CD4 T-cell recovery during ART. This finding appears paradoxical considering the role of IL-7 and IL-15 in supporting T-cell homeostatic proliferation [54], and data showing that exogenous IL-7 increased CD4 T-cell recovery when administered to people with HIV on ART [55]. Several factors may contribute to this result. Elevated levels of IL-7 and IL-15 concomitantly with lower CD4 cell count could result from lower IL-7 receptor expression on, or poor IL-7 responsiveness from [74] ISR CD4 T-cells. Although we had few samples available to perform *ex vitro* assays to determine the role of IL-7 and IL-15 cytokines, our results agree with previous studies, and show reduced IL-7 receptor expression in naïve, CM, and TM CD4 T-cell subsets at pre-ART and reduced activation and proliferation in response to IL-7 and IL-15 specifically for CM and TM T-cell subsets from ISR, including during ART. Thus, fewer CD4 T-cells available to bind circulating cytokines (lower consumption) can contribute, at least partially, to increased levels of IL-7 and IL-15 in ISRs. Of note, a recent study showed that phospholipase A2 group IB (PLA2G1B) synergized with the HIV gp41 envelope protein to inhibit CD4+ T-cell responses to IL-7 by promoting the formation of abnormal membrane microdomains which trap and inactivate IL-7R [75]. Furthermore, considering the key role of CM CD4 T-cells in maintaining homeostasis of the overall CD4 T-cell compartment [32], the higher levels of CM T-cells infection we described in ISR may prevent adequate repopulation of all CD4 memory cells despite higher cytokine levels. The presence of higher levels of IL-7 and IL-15 in ISR could contribute to the persistence of the HIV reservoir by increasing cell survival. Indeed, when administered to virally-suppressed participants (clinical trial NCT00099671), IL-7 led to a 70% increase in the absolute number of circulating CD4 T-cells harboring integrated HIV-DNA 4 weeks after therapy [76]. Intriguingly, our *ex vitro* study although limited to a small subset of patients available, suggests that CD4 T-cells from ISR respond to IL-7 and IL-15 stimulation by promoting the maintenance of a CM and TM T-cell phenotype and reducing the ability of those subsets to differentiate to EM CD4 T-cells. This scenario could indeed result in (i) higher maintenance of CM and TM T-cell subsets due to reduced differentiation to EM cells and (ii) a greater stability of the viral reservoir since CM and TM cells have a longer life-span and are more refractory to HIV reactivation [77] as compared to effector cells.

An important aspect of our analysis are the findings that provide a potential mechanism behind the differences in CD4 T-cell recovery and HIV persistence between ART-treated individuals. Expression of PD-1 and TIGIT in long-lived CD4 T-cell subsets appears to be the key determinant of baseline cellular viral load, post-ART responder status, and ultimate reservoir size, whereas LAG-3, TIM-3 and Ki67 were not predictive in our cohort. On the other hand, CD8 T-cell expression of PD-1 and TIGIT, or CD8 cytolytic potential do not differ between groups. Only EM CD8 T-cells express higher levels of granzyme B in ISRs compared with IRs at pre-ART. Although unlikely, aberrant CD8 killing of CD4 T-cells in ISRs at pre-ART could contribute to the higher decline in CD4 T-cell counts [78]. Separately, several studies have demonstrated the role of PD-1 to contribute to CD4 T-cell decline [79], T-cell dysfunction [80], suboptimal CD4 T-cell recovery [31,52], and greater proviral DNA burden [53], but this is the first study to provide a comprehensive illustration of how expression of this marker specifically on CM and TM CD4 T-cells at pre-ART is associated with hallmarks of HIV pathogenesis and persistence such as CD4 T-cell recovery and maintenance of the long-lived viral reservoir during ART. It is conceivable that targeting PD-1 and/or TIGIT expression especially early in disease could mitigate these downstream consequences.

Several aspects of this study design allowed for an unprecedented examination of ART response. First, it included samples before and at several years following ART initiation for the same participants. Furthermore, while the large majority of previous studies focused on bulk CD4 T-cells, our study focused on CD4 T-cell subsets. Finally, we were also able to compare the phenotype, frequency of infection, and response to homeostatic cytokines in different CD4 T-cell subsets between IR and ISR. There were several limitations to this study. Among the most important, pre-ART CD4 cell counts were significantly lower in ISR than IR and, since there are few IR with low baseline CD4 cell counts, the ability to adjust for this covariate was hampered. Furthermore, dichotomizing the outcomes (IR and ISR) could obscure more subtle findings than if a continuous measure was used instead. It is also important to note that measurement of total and integrated HIV-DNA overestimates the amount of replication competent HIV because >90% of the viruses quantified are defective [81]; due to the limited cell number in some of the purified subsets, we were not able to further define which frequency of those genomes are intact. Similarly, the number of samples available for *ex vitro* assays were limited, and further analysis could help to elucidate differences in IL-15/IL-7 responsiveness. Additionally, our analyses of Co-IRs did not include CTLA-4, a molecule recently indicated to identify a component of the persistent SIV and HIV reservoir even in absence of PD-1 [42]. Finally, we acknowledge that the findings observed between ISRs, and IRs in this study are associations and causality cannot be determined with the data available to date.

Improving survival and quality of life for people with HIV on-ART remains the most important goal for clinical programs around the world. CM and TM T-cell infection may hold the key to unlock further gains in extending quality adjusted life years for those individuals with ongoing inflammation and suboptimal CD4 T-cell recovery despite virologic suppression. Pushing the differentiation of CM and TM to EM T-cells or targeting their upregulation of PD-1 and TIGIT may be critical to design strategies aimed at improving immune reconstitution, reduce viral persistence, and promoting HIV remission.

## Materials and methods

### Ethics statement

This cohort was approved by the Emory University Institutional Review Board with approval number IRB00068326. Written consent was obtained from the participants.

### Study participants

Individuals with HIV were enrolled in a longitudinal cohort to study the effects of treatment on disease outcomes as part of the Emory University Center for AIDS Research (CFAR) specimen repository between 2010–2016. Baseline (pre-ART) and annual follow-up plasma and cell samples were stored for future study. Pre-ART samples were not available for all participants. Nineteen individual samples were available at pre-ART (IR: n = 10; ISR: n = 9) and 32 samples at on-ART time point (IR: n = 13; ISR: n = 19). Some of these cohort participants were recruited for an excisional inguinal LN biopsy on-ART based upon availability and the criteria listed in **S4 and S5 Table** (IR: n = 7; ISR: n = 7).

### Baseline measurements

Baseline was defined as prior to ART initiation. Participants in the studied cohort were not recruited at the time of infection. Thus, length of time of infection, peak viral load, and area under the viral load curve (AUC) are factors that could not be assessed. In this study, CD4 nadir is defined as the lowest CD4 T-cell count registered during the follow-up of the patient. Total and integrated HIV-DNA content, and markers of immune activation and cellular exhaustion (frequencies of CD38+, HLA DR+, Ki67+, PD-1+ and TIGIT+) measured in CD4 T-cell subsets were the baseline variables of interest. CD4 T-cell subsets were defined as naïve (N) CD45RA+CCR7+CD27+, central memory (CM) CD45RA-CCR7+CD27+, transitional memory (TM) CD45RA-CCR7-CD27+, and effector memory (EM) CD45RA-CCR7-CD27- CD4 T-cells.

### Study endpoints

The primary endpoint for CD4 T-cell recovery was achieving a CD4 cell count >500 cells/μL on or before two years after ART initiation (immune responders, IR) compared to individuals who did not achieve a CD4 cell count >500 cells/μL in the first two years after ART initiation (suboptimal immune responders, ISR) [30]. Inclusion for this endpoint required patients to be virologically suppressed. Sensitivity analyses also attempted to classify IRs/ISRs using the slope of CD4 T-cell recovery dichotomized at 100 cells per year based upon findings from a larger cohort study [19]. No significant results were observed. Similar to the baseline variables of interest, the endpoint variables were ongoing immune activation and cellular exhaustion, and total and integrated HIV-DNA of CD4 T-cell subsets.

### Sample processing

Blood samples were used within one hour of phlebotomy. PBMCs were isolated from whole blood by density gradient centrifugation. LN tissue was homogenized and passed through a 70-um cell strainer to isolate lymphocytes. All samples were processed, frozen in 10% dimethyl sulfoxide (DMSO) in heat-inactivated fetal bovine serum (FBS), and stored in liquid nitrogen until needed. Cells were thawed and washed twice in R10 [RPMI-1640 medium (Sigma-Aldrich) containing 10% FBS, and 10 U/ml DNase I (Roche Diagnostics)]. After final wash, the cells were resuspended in phosphate-buffered saline (PBS) and staining was performed.

### Flow cytometric analysis

Multi-parametric flow cytometric analysis was performed on peripheral blood and LN mononuclear cells according to standard procedures. Cell suspensions were stained with the viability dye LIVE/DEAD Aqua dye (Molecular probes) before being incubated with the following antibodies: anti-CD3-APC-Cy7 (clone SP34-2), anti-Ki67-Alexa700 (clone B56), anti-CCR7-PE-Cy7 (clone 3D12), anti-CD45RA-FITC (clone L48), anti-CCR5-APC (clone 3A9), anti-CD38-BV421 (clone HIT2), anti-HLA-DR-TRPE (clone G46-2), anti-CD4-BV650 (clone L200) all from BD Biosciences; anti-CD95-BV605 (clone DX2), anti-PD-1-PE (clone EH12.2H7), anti-TIM-3-BV605 (clone F38-2E2) from Biolegend; anti-LAG3-FITC (clone FAB2319) from R&D; anti-CD8-BV705 (clone 3B5) from Life technologies; anti-CD27-PE-Cy5 (clone 1A4CD27) from Beckman Coulter; anti-TIGIT-PerCP-Cy5.5 (clone MBSA43) from eBiosciences. After staining, cells were fixed (1% paraformaldehyde) and analyzed within 24 hours of collection. Flow cytometric acquisition was performed on at least 100,000 CD3+ T-cells on an LSRII cytometer (BD Biosciences) driven by the FACS DiVa software. Cell markers presenting less than 10,000 events acquired were excluded from the analyses. The data acquired were analyzed using FlowJo software (version 9.8.5; TreeStar). Analyses and graphical representation of Boolean combination of co-IRs were conducted using data analysis program SPICE (version 5.35) [82].

### FACS cell sorting

Mononuclear cells isolated from blood and LN tissue were stained with anti-CD3-APC-Cy7 (clone SP34-2), anti-CCR7-PE-Cy7 (clone 3D12), anti-CD45RA-FITC (clone L48), all from BD Biosciences; anti-CD8-BV705 (clone 3B5) from Life technologies; anti-CD27-PE-Cy5 (clone 1A4CD27) from Beckman Coulter; anti-CD4-BV421 (clone OKT4) from Biolegend. Different CD4+ T-cell subsets were then sorted (**S1 Fig**) based on their expression of CD45RA, CCR7 and CD27 using a FACS AriaII (BD Biosciences). Sorted CD4+ T-cell subsets were on average >96% pure as determined by post-sorting FACS analysis.

### IL-15 and IL-7 cytokine cell culture

CM, TM, and EM CD4 T-cell subsets were sorted from thawed PBMCs as explained above. Sorted cells were washed and resuspended (1 × 10^6^ cells per ml) in R10 with 30 ng/ml recombinant human IL-15 (R&D systems), and 30 ng/ml recombinant human IL-7 (R&D systems). Before and after five days of stimulation, cells were washed and stained with the following combination of monoclonal antibodies: anti-CCR7-BB700 (clone 3D12), anti-Ki67-AL700 (B56), anti-CD3-BUV395 (clone SP34-2), anti-CD45RA-BUV737 (clone HI100), anti-CD27-BV605 (clone L128), anti-CD8-BUV496 (clone RPA-T8), anti-granzyme-B-TRPE (clone GB11) all from BD Biosciences; anti-CD4-BV650 (clone OKT4), anti-HLA-DR-BV750 (clone L243), anti-PD-1-BV785 (clone EH12.2H7) all from Biolegend; anti-CD38-APC (clone OKT10) from Ray Biotech; anti-CD127-PE-Cy5 (clone EBIORDR5) from Scientific Thermofisher; anti-Perforin-FITC (clone Pf344) from MAbtech; LIVE/ DEAD Fixable Near-IR Dead Cell Stain from Thermo Fisher Scientific. Samples were collected on a FACSymphony system (BD Biosciences) and analyzed in FlowJo (version 9.9.6, TreeStar).

### Cell Trace proliferation

Cryo-preserved PBMCs were thawed, and washed with PBS. Cells were labeled with 5 μM CellTrace Violet (Thermo Fisher Scientific) in PBS at 37°C in a dark water bath for 20 minutes and quenched for 5 min over ice in the presence of 10% FBS. Cell Trace-labeled PBMCs were washed and resuspended (2.5 × 10^6^ cells per ml) in R10 with 30 ng/ml recombinant human IL-15, and 30 ng/ml recombinant human IL-7. Stimulated cells were fed with fresh medium supplemented with 60 ng/ml recombinant human IL-15, and 60 ng/ml recombinant human IL-7 (final concentration of 30 ng/ml for each cytokine) at day 4 and were harvested at day 7. After stimulation, cells were washed and stained with the following combination of monoclonal antibodies: anti-CD3-BUV395 (clone SP34-2), anti-CD8-BUV563 (clone RPA-T8), anti-Ki-67-AL700 (clone B56), all from BD Biosciences; anti-CD4-PerCP-Cy5.5 (clone OKT4) from Biolegend; LIVE/ DEAD Fixable Near-IR Dead Cell Stain from Thermo Fisher Scientific. Samples were collected on a FACSymphony system (BD Biosciences) and analyzed in FlowJo (version 9.9.6, TreeStar).

### Measurements of total and integrated HIV-DNA

Frequencies of CD4+ T-cells harboring total and integrated HIV-DNA were determined as previously described [83]. Briefly, cell pellets of sorted cells were resuspended in a lysis buffer (10 mM Tris-HCl pH 8.0, 50 nM KCl, 400 μg/mL Proteinase K, Invitrogen) and digested for 12–16 hours. Proteinase K was inactivated and cell lysates were directly used in all pre-amplification reactions. In all PCR reactions, primers specific for the human CD3 gene were used to quantify the exact number of cells present in the reaction tube. Total HIV-DNA or integrated HIV-DNA genomes (using *alu* oligonucleotides) together with the CD3 gene were pre-amplified for 12 cycles. The second rounds of PCR were carried out in real-time on the RotorGene Q instrument (Qiagen) with the Rotor Gene Probe Master Mix (Qiagen) following the manufacturer’s instructions. In all experiments, serial dilutions of digested ACH2 cells (containing more than a single copy of integrated HIV-DNA per cell when increases passage [84]) were used as standards for quantification. All measures of total and integrated HIV-DNA were performed in triplicate PCR reactions. Samples for which less than 3,000 cells were assayed were excluded from the analyses, and samples for which no target was detected are plotted at the limit of detection of the assay (calculated from cell input) and are represented as black symbols.

### Cytokine measurements

Cytokine expression profiles were evaluated by U-PLEX assay (Meso Scale MULTI-ARRAY Technology) commercially available by MSD. This technology allowed the evaluation of multiplexed biomarkers using custom made U-PLEX sandwich antibodies with a SULFO-TAG conjugated antibody and next generation of electrochemiluminescence (ECL) detection. An array of pro-inflammatory and anti-inflammatory cytokines were analyzed. The advantage of this platform is the broad dynamic range of detection (up to four logs—0.10 pg/mL to 1000 pg/mL). The assay was performed according to the manufacturer’s instructions. It required 25 μl of plasma from each donor combined with the biotinylated antibody plus the assigned linker and the SULFO-TAG conjugated detection antibody; in parallel a multi analyte calibrator standard was prepared by doing 4-fold serial dilutions. Both samples and calibrators were mixed with the Read buffer and loaded in a 10-spot U-PLEX plate, which was read by the MESO QuickPlex SQ 120. This instrument detects the electrochemiluminescence specific from each of the ten spots per well. The results were plotted in pg/mL and are extrapolated from the standard curve from each specific analyte.

### ELISA assay

To measure HIV-1 Env-specific IgG antibodies in patient plasma, 96-well plates (Nunc MaxiSorp, flat bottom Cat# 44-2404-21) were coated at 4°C overnight with 1 ng/μl gp120 (Con_B) (Immune Technology Cat# IT-001-CONBp) in carbonate-bicarbonate buffer (1.5 g Na2CO3 + 2.9 g NaHCO3 in 1 L H2O). The plates were washed 3x with PBS-T (0.05% Tween in PBS) and blocked for 2 hours with blocking buffer (3% BSA in PBS-T diluted from Blocker BSA 10% in PBS stock, Thermo Scientific Cat# 37525) followed by an additional 3 washes in PBS-T prior to the addition of samples. Patient plasma samples were heat inactivated at 56°C for 30 minutes and diluted in blocking buffer to starting concentrations between 1:100 and 1:1000. 100 μl of plasma was serially diluted in blocking buffer and added to the wells (eight concentrations tested for each sample) and incubated for two hours at room temperature. After six washes in PBS-T, HRP-conjugated anti-human IgG (Southern Biotech Cat# 2040–05) was added to each well at a 1:10,000 dilution in blocking buffer and incubated for 1h at room temperature. The plates were washed six times in PBS-T, and then tetramethylbenzidine SureBlue 1-component microwell peroxidase substrate (KPL Cat# 52-00-03) was added. The plate was incubated in the dark at room temperature for 10 minutes, at which time 4 N H2SO4 stop solution (Fisher Scientific Cat # SA818-1) was added. The optical density at 450 nm was determined on a Cytation 3 Cell Imaging Multimode Reader (BioTek), and the data were analyzed using Gen5 software. Quantitation of Env-specific plasma IgG was performed by including a standard curve for human IgG on each plate. For this, two columns of each plate were treated as above except for coating with Goat Anti-Human IgG (Southern Biotech Cat# 2015–01) and incubating with serial dilutions of human IgG (Southern Biotech CAT 0150–01; seven dilutions in blocking buffer starting at 100 ng/ml). Concentrations of plasma IgG that bound to gp120 were determined using all dilutions that fell within the range of the standard curve and were represented by the average for each sample. Each assay was run with duplicate wells and one independent repeat.

### Statistical analysis

Repeated-measures analyses for longitudinal continuous outcomes (i.e. CD4 T-cell subsets) were performed with a means model via the SAS MIXED Procedure (version 9.4; SAS Institute, Cary, NC), providing separate estimates of the means by time on study (Pre-ART and on-ART) and IR group. Repeated-measures analyses allow to study the longitudinal correlation between multiple observations from the same individual. Repeated-measures analyses were implemented using a linear mixed effects model, due to its flexibility and efficiency for statistical inference. The model included three predictors (IR group, time on study and the statistical interaction between IR group and time on study). This model estimates the mean and the variances (within-individual and between-individual) for each outcome and allows tests to estimate the effect of study group (IR or ISR) at each follow-up assessment, and to obtain average effects across the time assessments. A compound-symmetric variance-covariance form in repeated measurements was assumed for each outcome and robust estimates of the standard errors of parameters were used to perform statistical tests and construct 95% confidence intervals [85]. The model-based means are unbiased with unbalanced and missing data, so long as the missing data are non-informative (missing at random, MAR). A P-value ≤0.05 was considered statistically significant for the main effects (IR group and time on study) and for the IR group by time on study interaction effect from the repeated measures analysis. The statistical test for interaction between time on study and the IR group was the primary overall hypothesis test to determine whether each outcome in the two study groups changed in significantly different ways during follow-up (i.e., different temporal patterns over time). If an outcome in the two study groups was consistently different or similar over time (i.e., no statistical interaction) then the main effect test for study group was used as the primary test. If a significant interaction was detected, then t-tests were used to compare the differences between the model-based IR group means at each time point and to compare differences over time within each study group. Specific statistical tests were done within the framework of the mixed effects linear model. All statistical tests were 2-sided and unadjusted for multiple comparisons.

Rate of decline for total and integrated HIV-DNA decay in CD4 T-cells (log_10_ transformed) was calculated using a mixed-effects linear model specifying that each outcome follows a linear regression over time with a random intercept for each participant. The slope analysis is valid under the assumption of missing at random (MAR; i.e. the missing data mechanism is dependent only on the observed data).

Mean changes of unpaired observations were assessed using the Mann Whitney u-test, which was considered significant at *P* < 0.05. Spearman rank correlation tests were used as specified. Analyses and Figs were generated with the GraphPad Prism v5.0b Software.

## Supporting information

S1 Fig Gating strategy of different T-cell subsets including naïve (N), central memory (CM), transitional memory (TM), and effector memory (EM) cells. CD38, HLA-DR, Ki67, PD-1, TIGIT representative staining inside CM CD4 T-cell subset is shown in the bottom.
(TIF)

S2 Fig ISR present higher frequencies of CD8 EM subset at pre-, and on-ART in blood, but no differences in lymph node. (A, and B) Levels of peripheral blood CD8 T-cell subsets before (IR, n = 10; ISR, n = 9) (A) and after (IR, n = 12; ISR, n = 19) (B) ART initiation in immunologic responder (IR; blue) and suboptimal responder (ISR; red). (C, and D) Levels of lymph node (LN) CD4 T-cell (C), and CD8 T-cell (D) subsets after ART initiation in IR (n = 7) and ISR (n = 7). Data show mean values and SEM. Repeated-measures analyses were performed with a means model (SAS MIXED Procedure, version 9.4) to generate statistical outcomes between IR and ISR individuals.
(TIF)

S3 Fig The frequency of blood and lymph node CD4 T-cells expressing one or more Co-IRs is higher in ISR as compared to IR. (A, and B) Bar and pie chart representation of co-inhibitory receptors (co-IRs) expression in peripheral blood total CD4 T-cells before (IR, n = 10; ISR, n = 9) (A) and after (IR, n = 12; ISR, n = 19) (B) ART initiation among immunologic responder (IR) and suboptimal responder (ISR) participants. (C) Bar and pie chart representation of lymph node (LN) co-inhibitory receptors (co-IRs) expression in total CD4 T-cells after ART initiation among IR (n = 7), and ISR (n = 7) participants. Analysis of co-IRs expression was performed using a Boolean gating strategy in SPICE. Each slice of the pie charts represents the proportion of a combination of co-IRs: black 4 co-IRs, dark blue 3 co-IRs, marine blue 2 co-IRs, light blue 1 co-IR, and grey no co-IRs. Bar charts show the frequency of each population with bars representing median co-IRs expressions (IR, blue bars and ISR, red bars). Statistical differences between IR and ISR, participants living with HIV are indicated above pie charts and calculated with SPICE software.
(TIF)

S4 Fig PD-1 and TIGIT expression levels are higher in ISR as compared to IR in blood and LN. **A-D.** Frequency of co-inhibitory receptors (co-IRs), PD-1 (A), TIGIT (B), LAG-3 (C) and TIM-3 (D) levels of peripheral blood total CD4 T-cells before (IR, n = 10; ISR, n = 9), and after ART (IR, n = 12; ISR, n = 19) initiation among immunologic responders (IR; blue) and suboptimal responders (ISR; red). (E-H) Frequency of co-inhibitory receptors (co-IRs), PD-1 (E), TIGIT (F), LAG-3 (G) and TIM-3 (H) levels of lymph node (LN) total CD4 T-cells after ART initiation among IR (n = 7) and ISR (n = 7) participants. Data show mean values and SEM. Mann Whitney u-test was used to compare differences between IR and ISR individuals.
(TIF)

S5 Fig CD8 T-cell subsets differences in co-IRs, and Ki67 expression between IR and ISR. Co-inhibitory receptors (co-IRs), PD-1 (A and D) and TIGIT (B and E), and proliferation Ki67 (C and F) levels of peripheral blood CD8 T-cell subsets before (IR, n = 10; ISR, n = 9) (A-C) and after (IR, n = 12; ISR, n = 19) (D-F) ART initiation among immunologic responder (IR; blue) and suboptimal responder (ISR; red) participants. CD8 T-cell subsets included naïve (N), effector memory (EM), transitional memory (TM), and central memory (CM) cells. Data show mean values and SEM. Repeated-measures analyses were performed with a means model (SAS MIXED Procedure, version 9.4) to generate statistical outcomes between IR and ISR individuals.
(TIF)

S6 Fig Levels of T-cell exhaustion markers on peripheral blood CD4 T-cell subsets before ART initiation in immunologic responder, and non-responder, individuals living with HIV. (A) Expression of T-cell exhaustion markers PD-1, and (B) TIGIT on blood CD4 T-cell CM subset before ART initiation in immunologic responders (IR; blue, n = 9) and non-responders (INR; red, n = 4). Threshold to define INR is of <350 CD4 cells/μL ≥2 years on-ART. Data show mean values and SEM. Mann Whitney u-test was used to compare differences between IR and INR individuals.
(TIF)

S7 Fig Co-IR expression and HIV-DNA levels of T-cell subsets on lymph node of IR and ISR during ART. (A, D) PD-1, (B, E) TIGIT, and (C, F) Ki67 levels of lymph node (LN) CD4 T-cell (A-C), and CD8 T-cell (D-F) subsets after ART initiation among immunologic responder (IR; blue, n = 7) and suboptimal responder (ISR; red, n = 7) participants. (G) Total HIV-DNA cellular infection in LN CD4 T-cell subsets after ART initiation (IR, n = 5; ISR, n = 7). (H) Integrated HIV-DNA cellular infection in LN CD4 T-cell subsets after ART initiation (IR, n = 6; ISR, n = 7). T-cell subsets included naïve (N), effector memory (EM), transitional memory (TM), and central memory (CM) cells. Data show mean values and SEM. Repeated-measures analyses were performed with a means model (SAS MIXED Procedure, version 9.4) to generate statistical outcomes between IR and ISR individuals.
(TIF)

S8 Fig Total and integrated HIV-DNA log_10_ copies corrected per absolute counts. HIV-DNA (Log_10_ copies/1*10^6^ cells) was corrected per absolute cell counts (cells/mm^3^ blood) for each CD4 T-cell subset in IR (blue) vs ISR (red) at pre- (A, and B), and on- ART (C, and D). Data show mean values and SEM. Mann Whitney u-test was used to compare differences between IR and ISR individuals.
(TIF)

S9 Fig HIV-DNA levels on peripheral blood CD4 T-cell subsets after ART initiation in immunologic responder, and non-responder, individuals living with HIV. (A) Total HIV-DNA cellular infection in peripheral blood CD4 T-cell subsets after ART initiation among immunologic responder (IR; blue, n = 12) and non-responder (INR; red, n = 11) participants. (B) Integrated HIV-DNA cellular infection in peripheral blood CD4 T-cell subsets after ART initiation among IR and INR, participants living with HIV. Threshold to define INR is of <350 CD4 cells/μL ≥2 years on-ART. Data show mean values and SEM. Mann Whitney u-test was used to compare differences between IR and INR individuals.
(TIF)

S10 Fig ISR present a greater reduction than IR in total and integrated HIV-DNA levels for naïve cells. (A) Percentage of reduction from pre-ART of total and (B) integrated HIV-DNA on naïve CD4 T-cells on immunologic (IR; blue bar) and suboptimal immunologic responders (ISR; red bar). Data show mean values and SEM. Statistical differences were assessed with a one sample t-test.
(TIF)

S11 Fig PD-1 and TIGIT expression on CM CD4 T-cells is correlated with a decline in the CD4 slope. Correlations between co-inhibitory receptors (co-IRs), PD-1 (A) and TIGIT (B), levels in CM CD4 T-cells at pre-ART and CD4 T-cell recovery (slope; cells/mm^3^) in immunologic responder (IR; blue, n = 10) and suboptimal responder (ISR; red, n = 9) participants. Spearman rank correlation test was used to determine correlations among all individuals.
(TIF)

S12 Fig IL-7+IL-15 don’t differently impact HIV DNA and RNA levels in sorted CD4 T-cells IRs and ISRs. (A) HIV-RNA supernatant, and (B) cell-associated HIV-DNA of sorted CD4 T-cell subsets after 5 days of IL-15+IL7 stimulation from on-ART ISR (red, n = 3) and IR (blue, n = 4) individuals. CD4 T-cell subsets included naïve (N), central memory (CM), transitional memory TM, and effector memory (EM) cells. Black symbols correspond to undetectable values. Averaged data are presented as the mean ± SEM. Mann Whitney u-test was used for statistical analysis.
(TIF)

S1 Table Demographic and clinical characteristics of the study participants. (DOCX)

S2 Table List of cytokines measured in plasma at pre-ART time point. Data show statistical differences between IR and ISR. SD, standard deviation.
(DOCX)

S3 Table Raw means and 95% confidence intervals for HIV-DNA levels in blood (PBMC) CD4 T-cell subsets (Naïve, CM, TM and EM). (DOCX)

S4 Table Inclusion and exclusion criteria for participation in the lymph node biopsy on-ART. The total of LN biopsies available were 14 total, 7 for immunological responders (IR), and 7 for immunological suboptimal responders (ISR).
(DOCX)

S5 Table Demographic and clinical characteristics of the study participants with lymph node biopsy on-ART available. (DOCX)

S6 Table Raw flow cytometry data. (XLSX)
