# Supplementary figures and images for: Increased homeostatic cytokines and stability of HIV-infected memory CD4 T-cells identify individuals with suboptimal CD4 T-cell recovery on-ART
Source: PLoS Pathog. 2021 Aug 27;17(8):e1009825. doi: 10.1371/journal.ppat.1009825 (PMC8397407; doi:10.1371/journal.ppat.1009825)

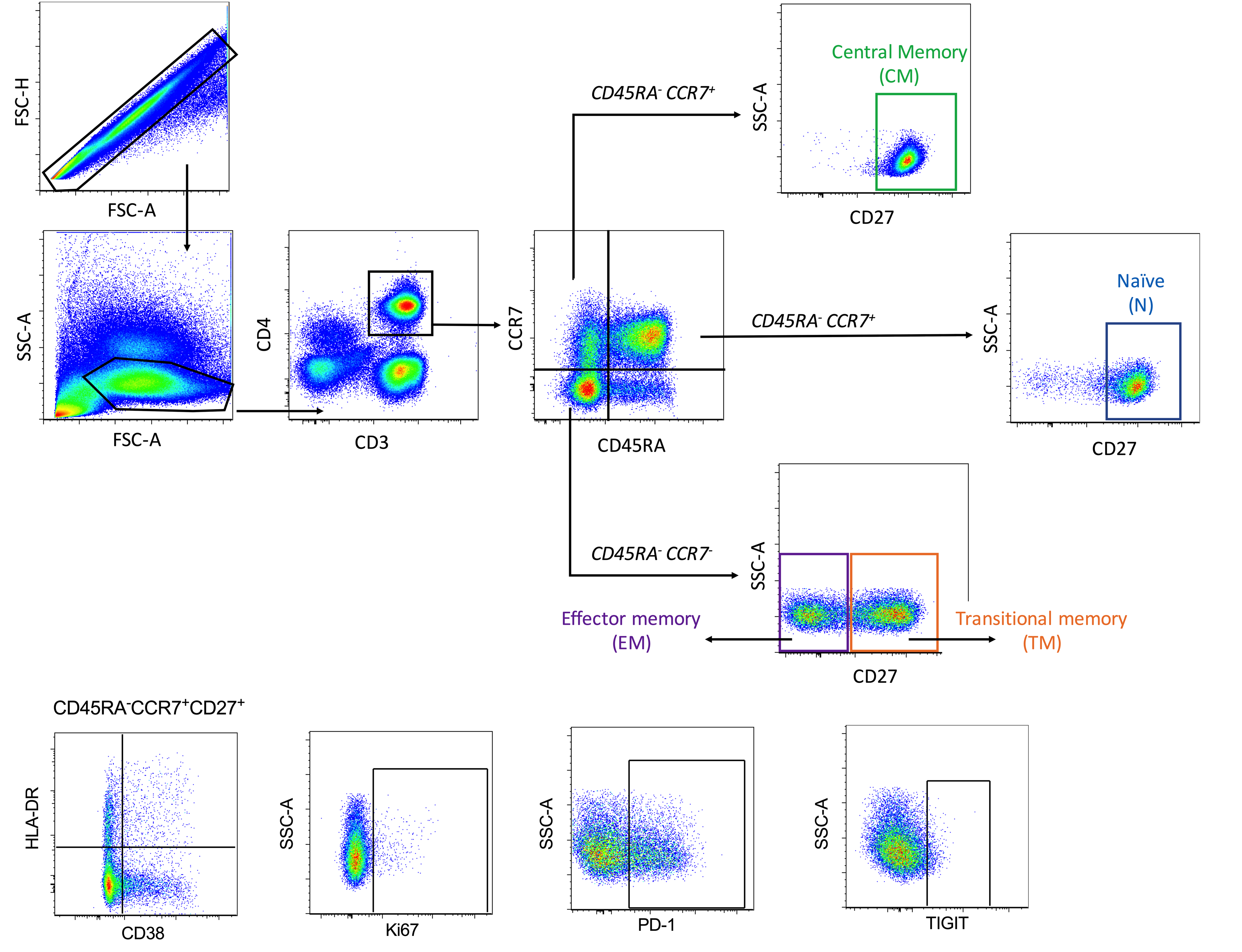

Supplement: S1 Fig — CD38, HLA-DR, Ki67, PD-1, TIGIT representative staining inside CM CD4 T-cell subset is shown in the bottom. (TIF) [file ppat.1009825.s001.tif]

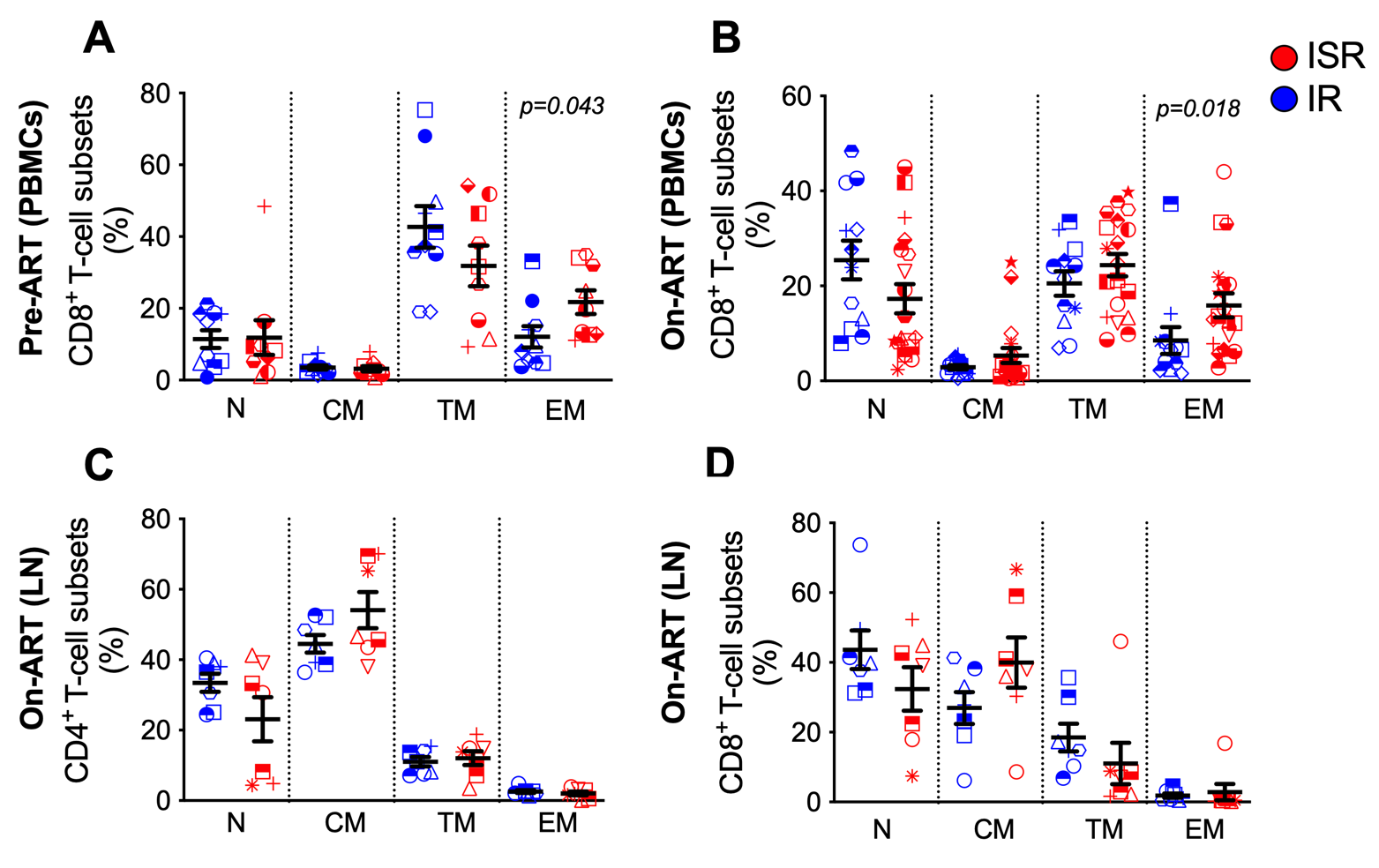

Supplement: S2 Fig — (A, and B) Levels of peripheral blood CD8 T-cell subsets before (IR, n = 10; ISR, n = 9) (A) and after (IR, n = 12; ISR, n = 19) (B) ART initiation in immunologic responder (IR; blue) and suboptimal responder (ISR; red). (C, and D) Levels of lymph node (LN) CD4 T-cell (C), and CD8 T-cell (D) subsets after ART initiation in IR (n = 7) and ISR (n = 7). Data show mean values and SEM. Repeated-measures analyses were performed with a means model (SAS MIXED Procedure, version 9.4) to generate statistical outcomes between IR and ISR individuals. (TIF) [file ppat.1009825.s002.tif]

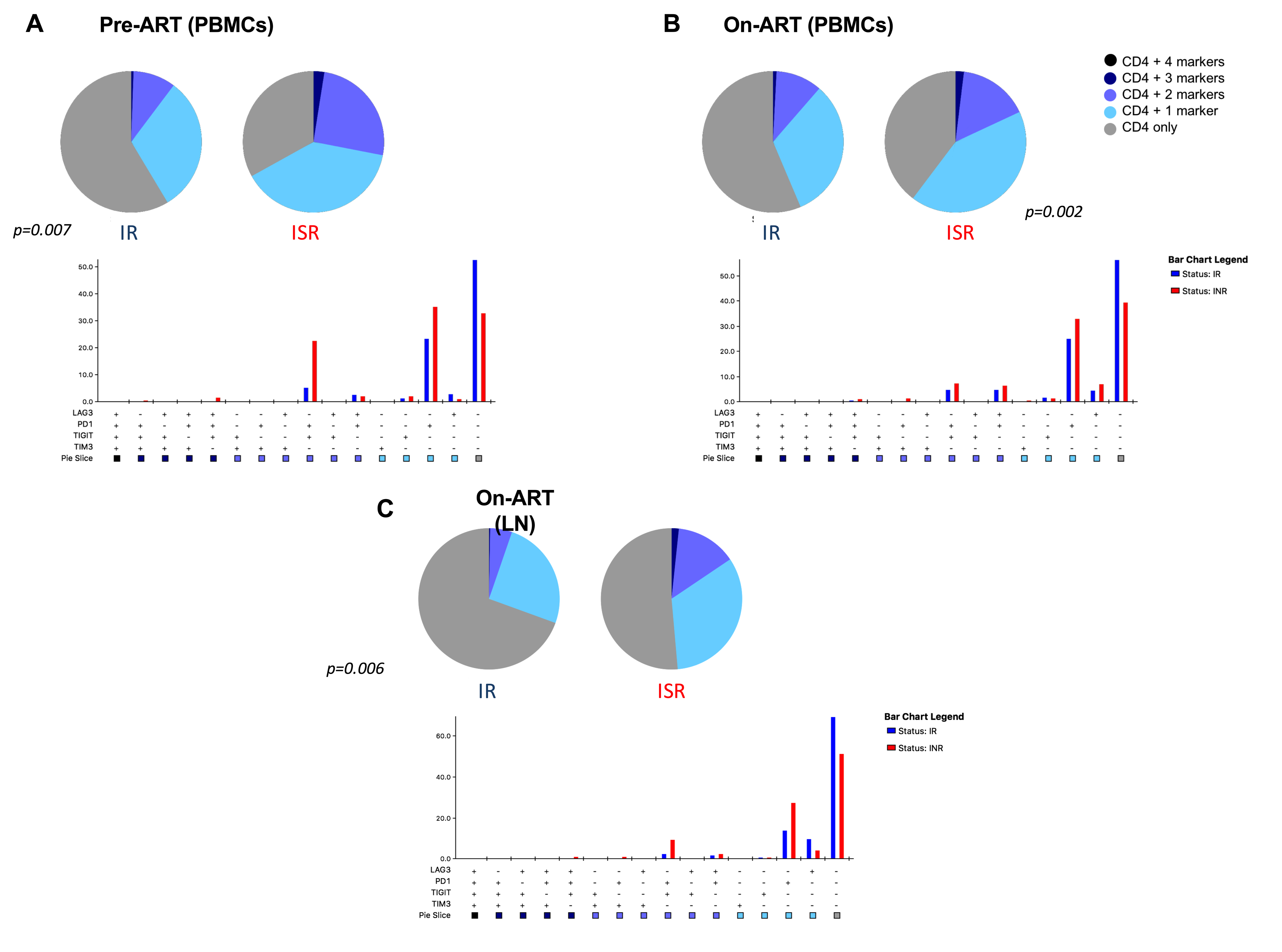

Supplement: S3 Fig — (A, and B) Bar and pie chart representation of co-inhibitory receptors (co-IRs) expression in peripheral blood total CD4 T-cells before (IR, n = 10; ISR, n = 9) (A) and after (IR, n = 12; ISR, n = 19) (B) ART initiation among immunologic responder (IR) and suboptimal responder (ISR) participants. (C) Bar and pie chart representation of lymph node (LN) co-inhibitory receptors (co-IRs) expression in total CD4 T-cells after ART initiation among IR (n = 7), and ISR (n = 7) participants. Analysis of co-IRs expression was performed using a Boolean gating strategy in SPICE. Each slice of the pie charts represents the proportion of a combination of co-IRs: black 4 co-IRs, dark blue 3 co-IRs, marine blue 2 co-IRs, light blue 1 co-IR, and grey no co-IRs. Bar charts show the frequency of each population with bars representing median co-IRs expressions (IR, blue bars and ISR, red bars). Statistical differences between IR and ISR, participants living with HIV are indicated above pie charts and calculated with SPICE software. (TIF) [file ppat.1009825.s003.tif]

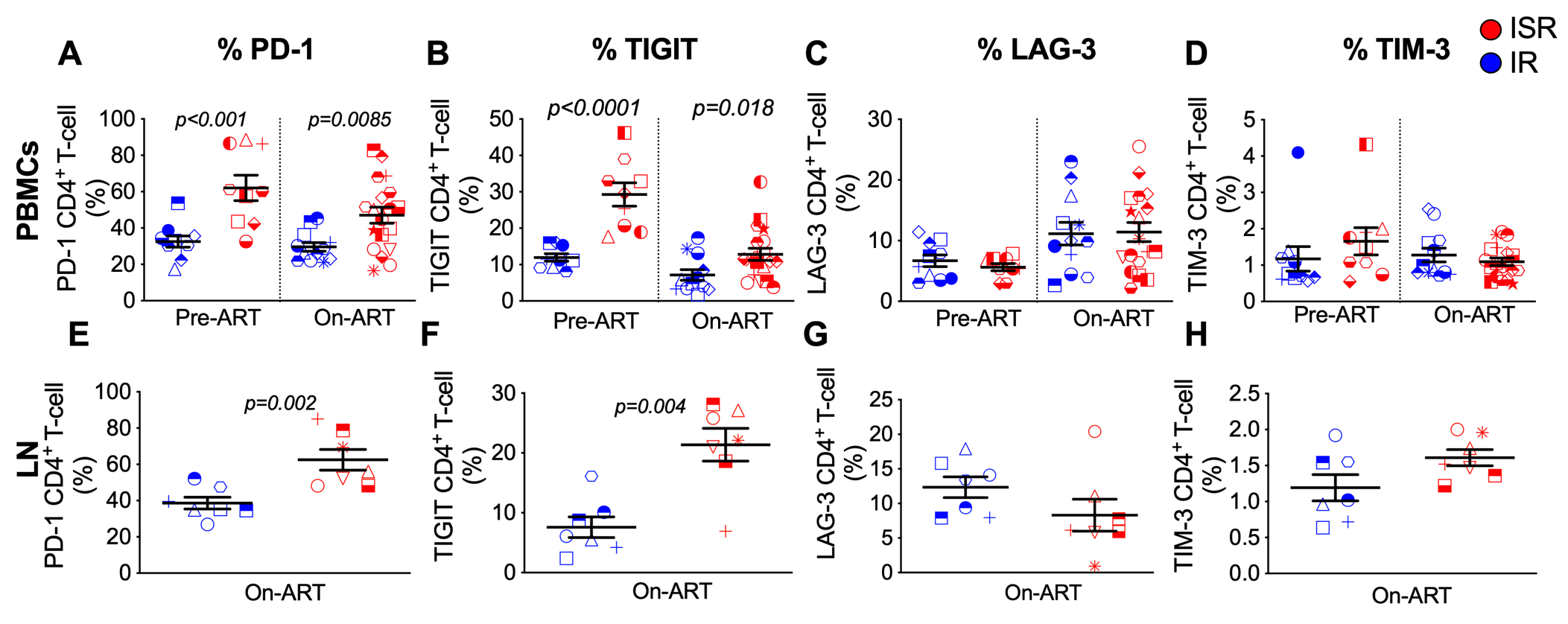

Supplement: S4 Fig — A-D. Frequency of co-inhibitory receptors (co-IRs), PD-1 (A), TIGIT (B), LAG-3 (C) and TIM-3 (D) levels of peripheral blood total CD4 T-cells before (IR, n = 10; ISR, n = 9), and after ART (IR, n = 12; ISR, n = 19) initiation among immunologic responders (IR; blue) and suboptimal responders (ISR; red). (E-H) Frequency of co-inhibitory receptors (co-IRs), PD-1 (E), TIGIT (F), LAG-3 (G) and TIM-3 (H) levels of lymph node (LN) total CD4 T-cells after ART initiation among IR (n = 7) and ISR (n = 7) participants. Data show mean values and SEM. Mann Whitney u-test was used to compare differences between IR and ISR individuals. (TIF) [file ppat.1009825.s004.tif]

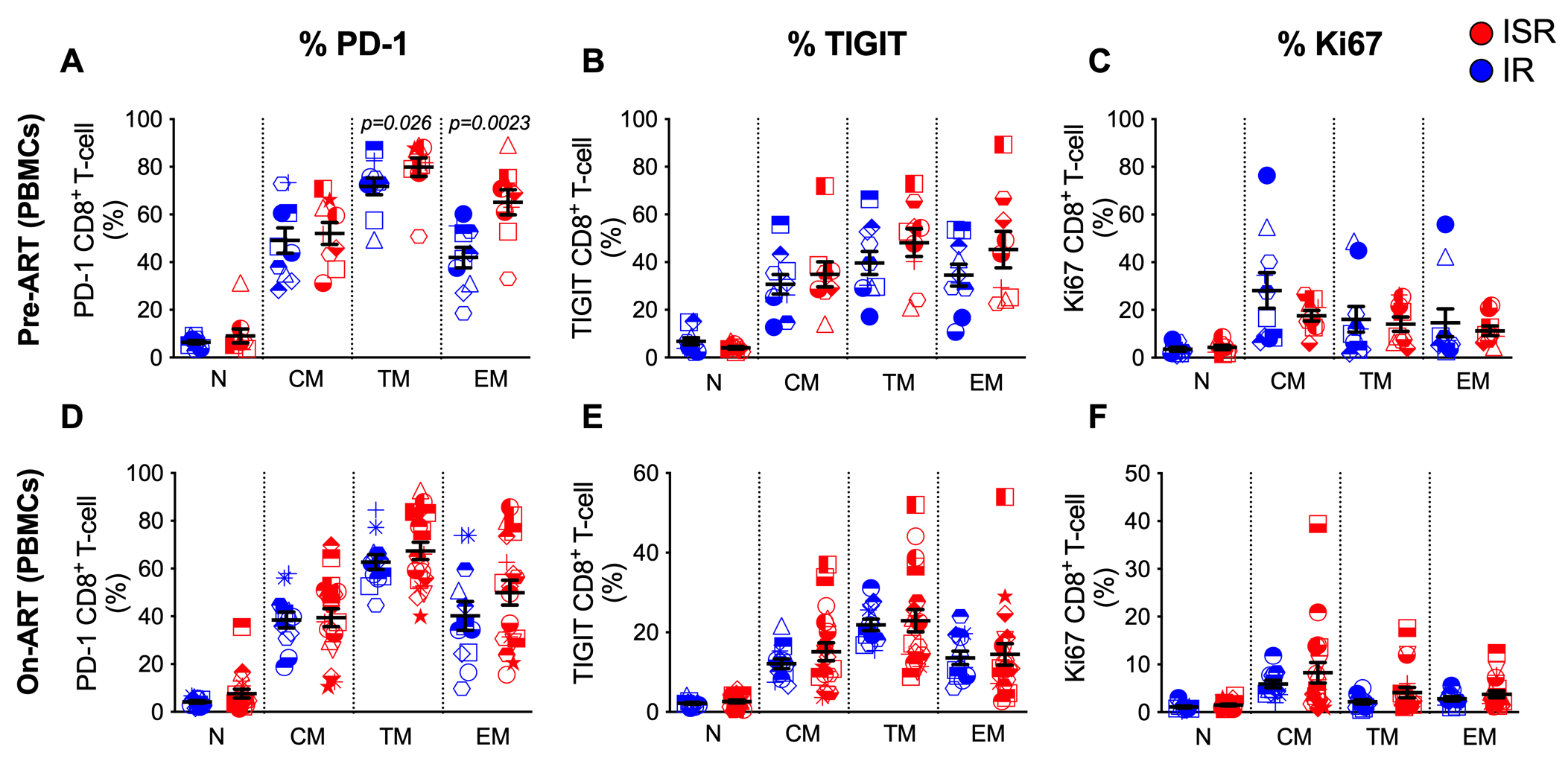

Supplement: S5 Fig — Co-inhibitory receptors (co-IRs), PD-1 (A and D) and TIGIT (B and E), and proliferation Ki67 (C and F) levels of peripheral blood CD8 T-cell subsets before (IR, n = 10; ISR, n = 9) (A-C) and after (IR, n = 12; ISR, n = 19) (D-F) ART initiation among immunologic responder (IR; blue) and suboptimal responder (ISR; red) participants. CD8 T-cell subsets included naïve (N), effector memory (EM), transitional memory (TM), and central memory (CM) cells. Data show mean values and SEM. Repeated-measures analyses were performed with a means model (SAS MIXED Procedure, version 9.4) to generate statistical outcomes between IR and ISR individuals. (TIF) [file ppat.1009825.s005.tif]

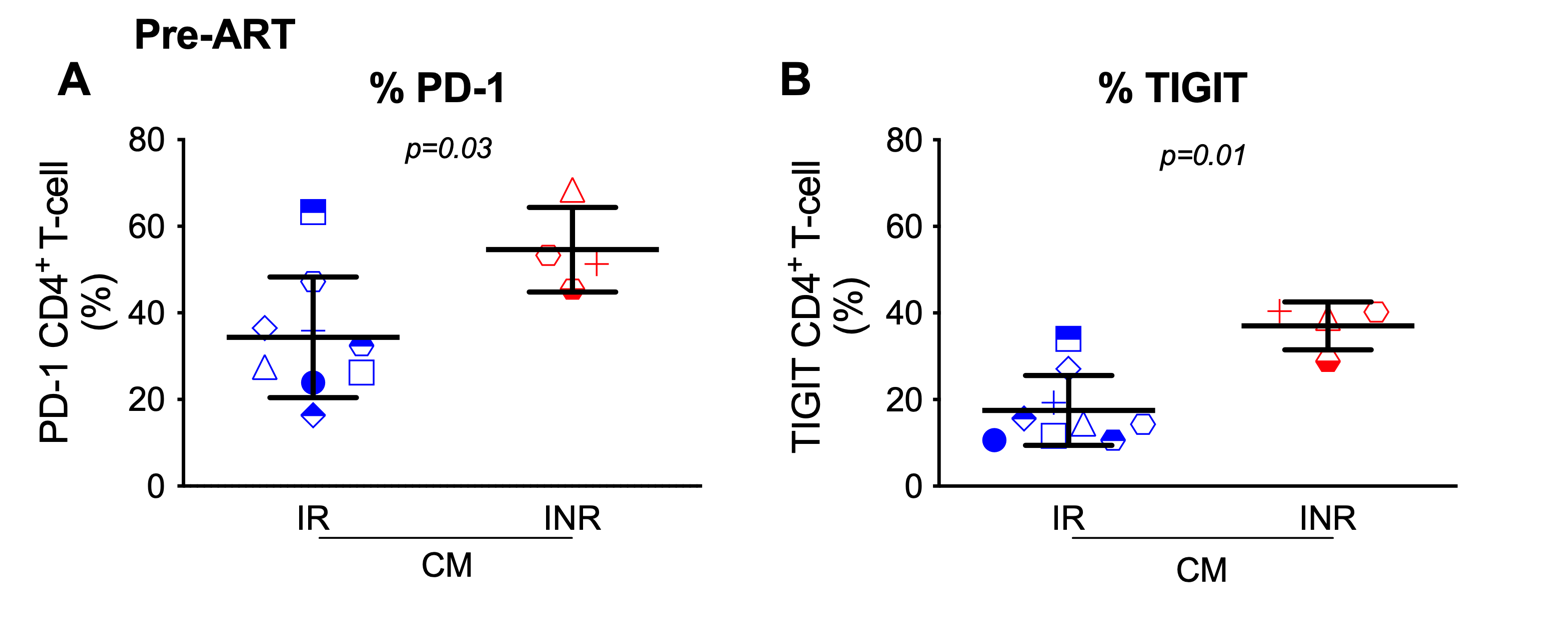

Supplement: S6 Fig — (A) Expression of T-cell exhaustion markers PD-1, and (B) TIGIT on blood CD4 T-cell CM subset before ART initiation in immunologic responders (IR; blue, n = 9) and non-responders (INR; red, n = 4). Threshold to define INR is of <350 CD4 cells/μL ≥2 years on-ART. Data show mean values and SEM. Mann Whitney u-test was used to compare differences between IR and INR individuals. (TIF) [file ppat.1009825.s006.tif]

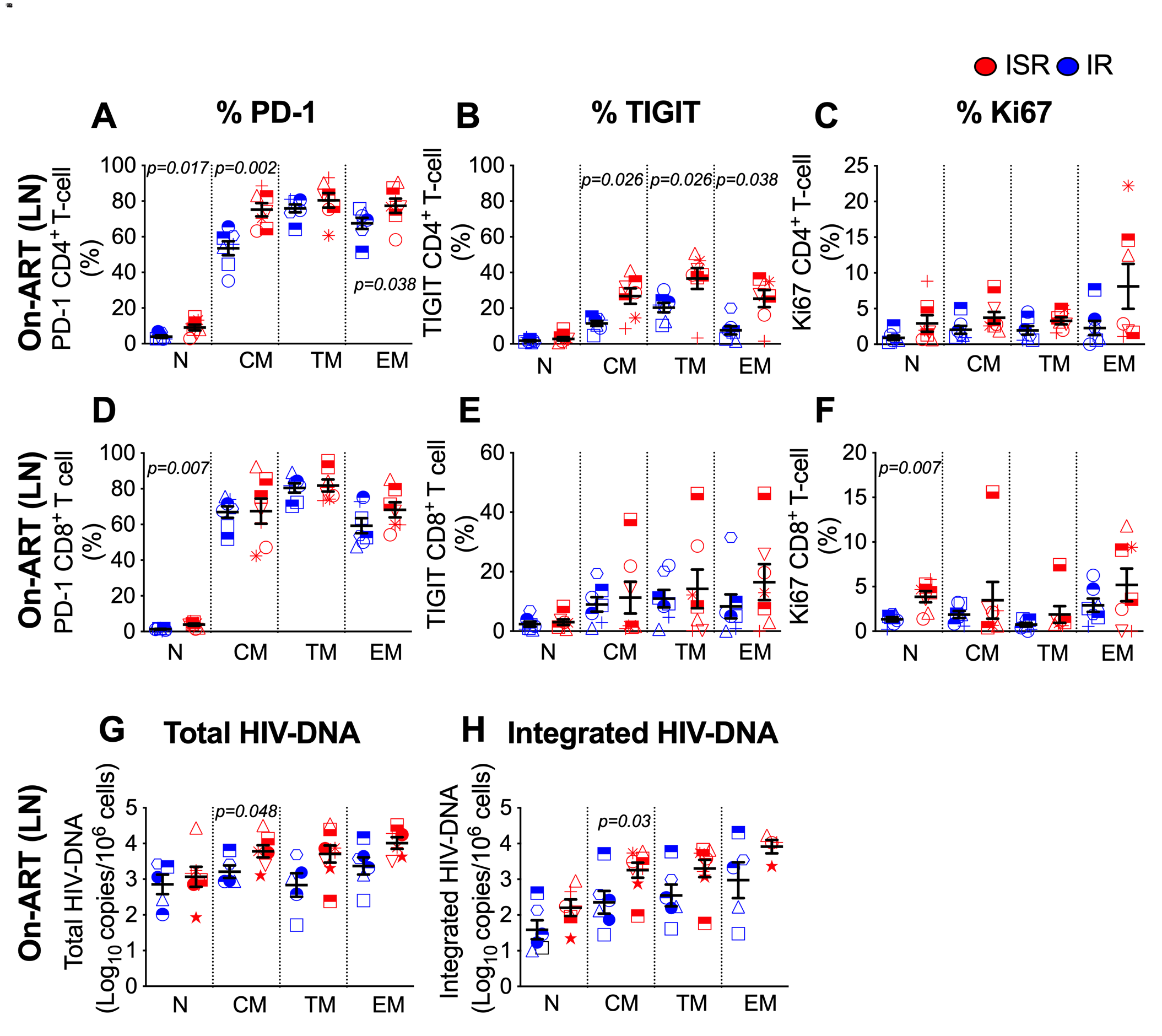

Supplement: S7 Fig — (A, D) PD-1, (B, E) TIGIT, and (C, F) Ki67 levels of lymph node (LN) CD4 T-cell (A-C), and CD8 T-cell (D-F) subsets after ART initiation among immunologic responder (IR; blue, n = 7) and suboptimal responder (ISR; red, n = 7) participants. (G) Total HIV-DNA cellular infection in LN CD4 T-cell subsets after ART initiation (IR, n = 5; ISR, n = 7). (H) Integrated HIV-DNA cellular infection in LN CD4 T-cell subsets after ART initiation (IR, n = 6; ISR, n = 7). T-cell subsets included naïve (N), effector memory (EM), transitional memory (TM), and central memory (CM) cells. Data show mean values and SEM. Repeated-measures analyses were performed with a means model (SAS MIXED Procedure, version 9.4) to generate statistical outcomes between IR and ISR individuals. (TIF) [file ppat.1009825.s007.tif]

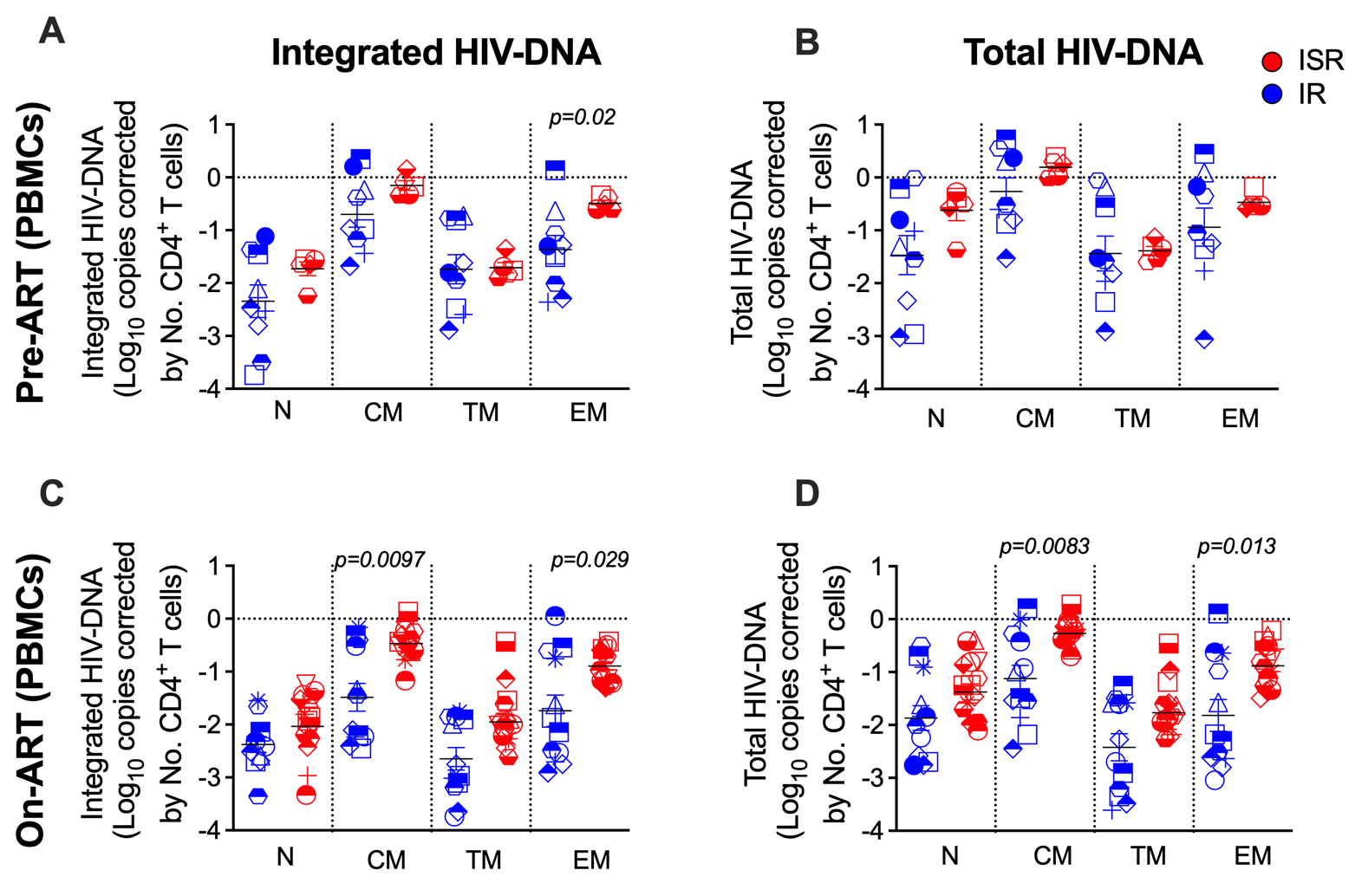

Supplement: S8 Fig — HIV-DNA (Log10 copies/1*106 cells) was corrected per absolute cell counts (cells/mm3 blood) for each CD4 T-cell subset in IR (blue) vs ISR (red) at pre- (A, and B), and on- ART (C, and D). Data show mean values and SEM. Mann Whitney u-test was used to compare differences between IR and ISR individuals. (TIF) [file ppat.1009825.s008.tif]

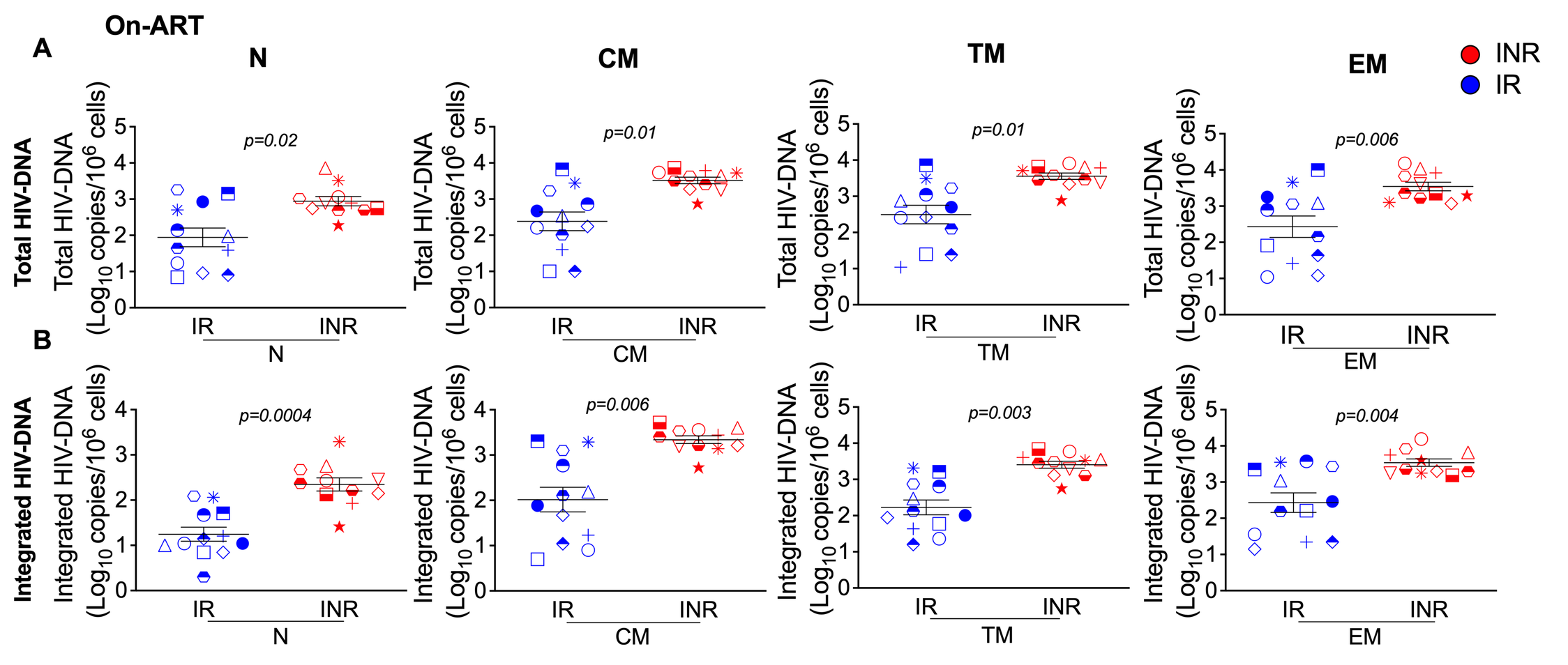

Supplement: S9 Fig — (A) Total HIV-DNA cellular infection in peripheral blood CD4 T-cell subsets after ART initiation among immunologic responder (IR; blue, n = 12) and non-responder (INR; red, n = 11) participants. (B) Integrated HIV-DNA cellular infection in peripheral blood CD4 T-cell subsets after ART initiation among IR and INR, participants living with HIV. Threshold to define INR is of <350 CD4 cells/μL ≥2 years on-ART. Data show mean values and SEM. Mann Whitney u-test was used to compare differences between IR and INR individuals. (TIF) [file ppat.1009825.s009.tif]

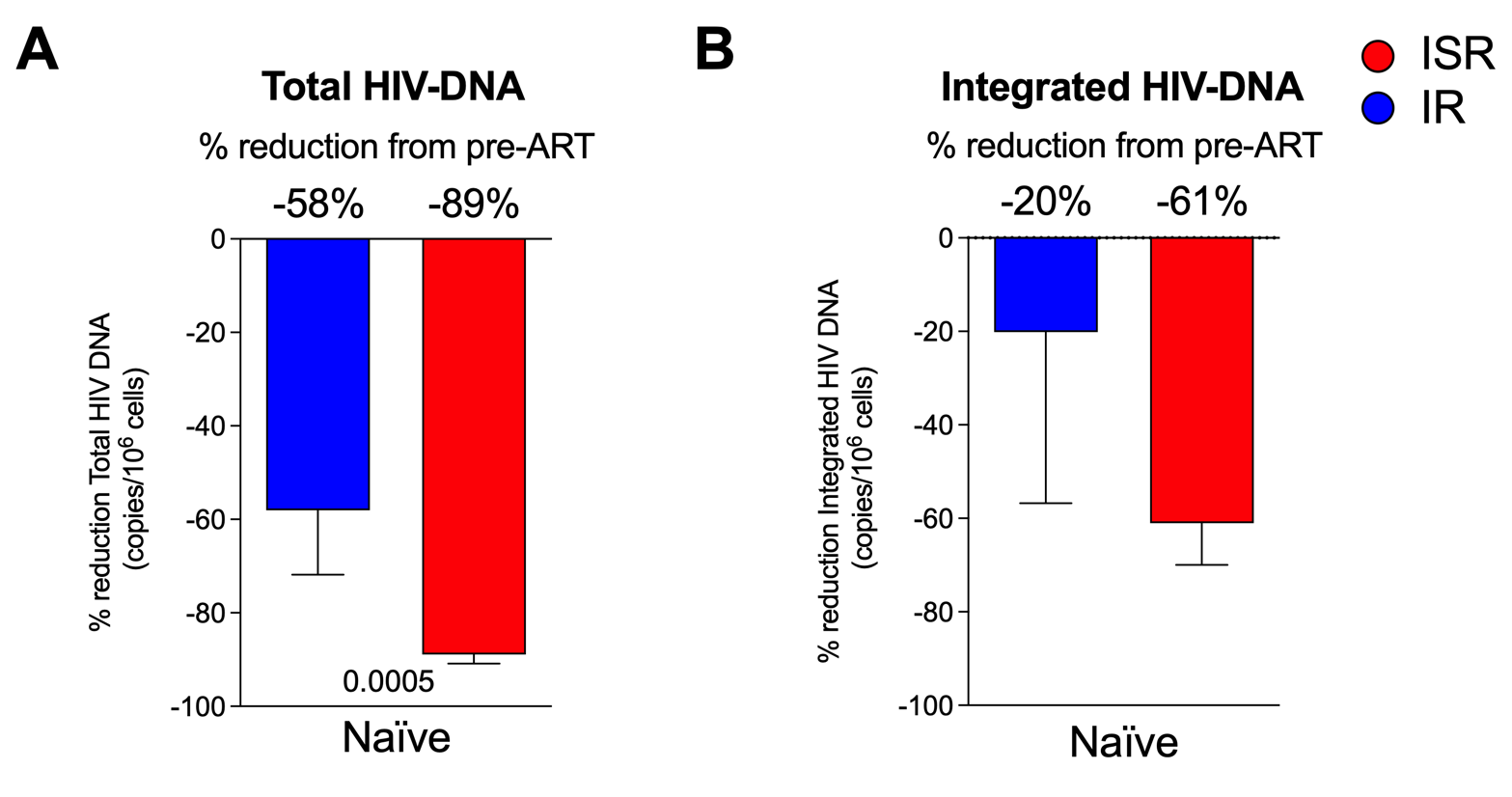

Supplement: S10 Fig — (A) Percentage of reduction from pre-ART of total and (B) integrated HIV-DNA on naïve CD4 T-cells on immunologic (IR; blue bar) and suboptimal immunologic responders (ISR; red bar). Data show mean values and SEM. Statistical differences were assessed with a one sample t-test. (TIF) [file ppat.1009825.s010.tif]

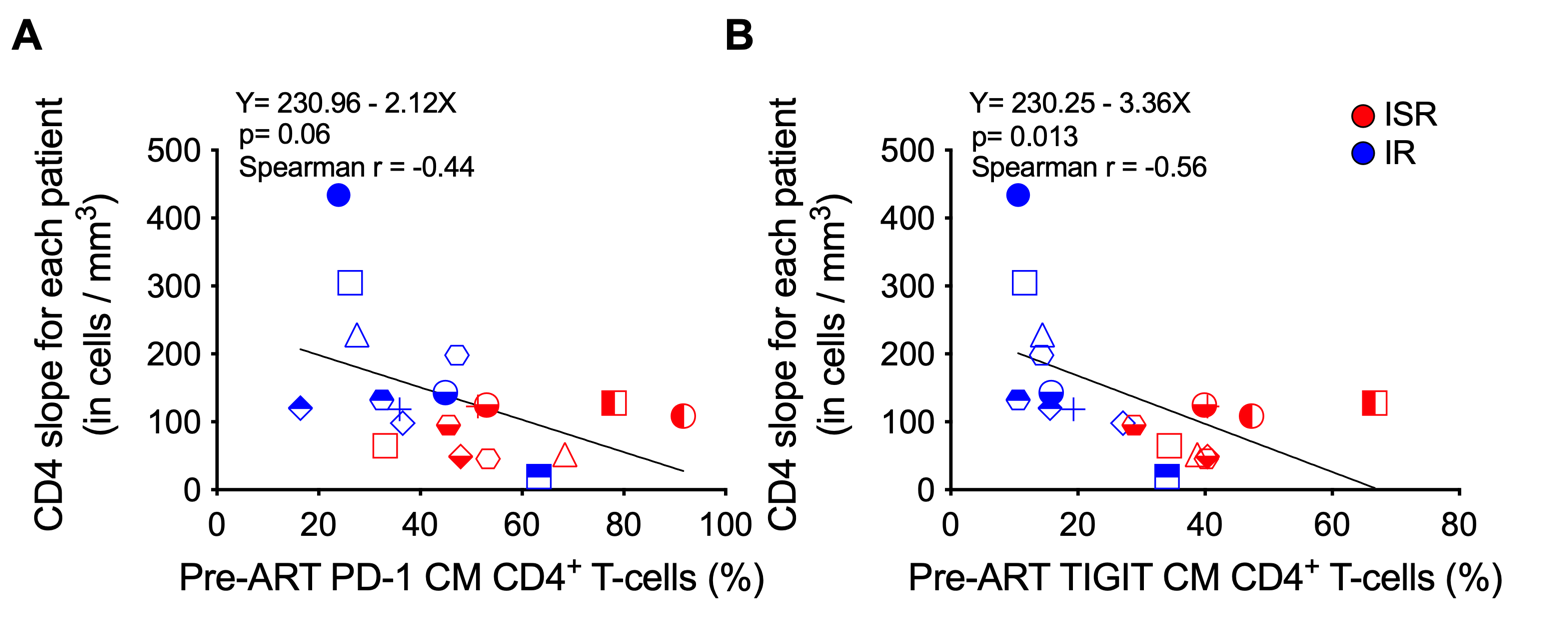

Supplement: S11 Fig — Correlations between co-inhibitory receptors (co-IRs), PD-1 (A) and TIGIT (B), levels in CM CD4 T-cells at pre-ART and CD4 T-cell recovery (slope; cells/mm3) in immunologic responder (IR; blue, n = 10) and suboptimal responder (ISR; red, n = 9) participants. Spearman rank correlation test was used to determine correlations among all individuals. (TIF) [file ppat.1009825.s011.tif]

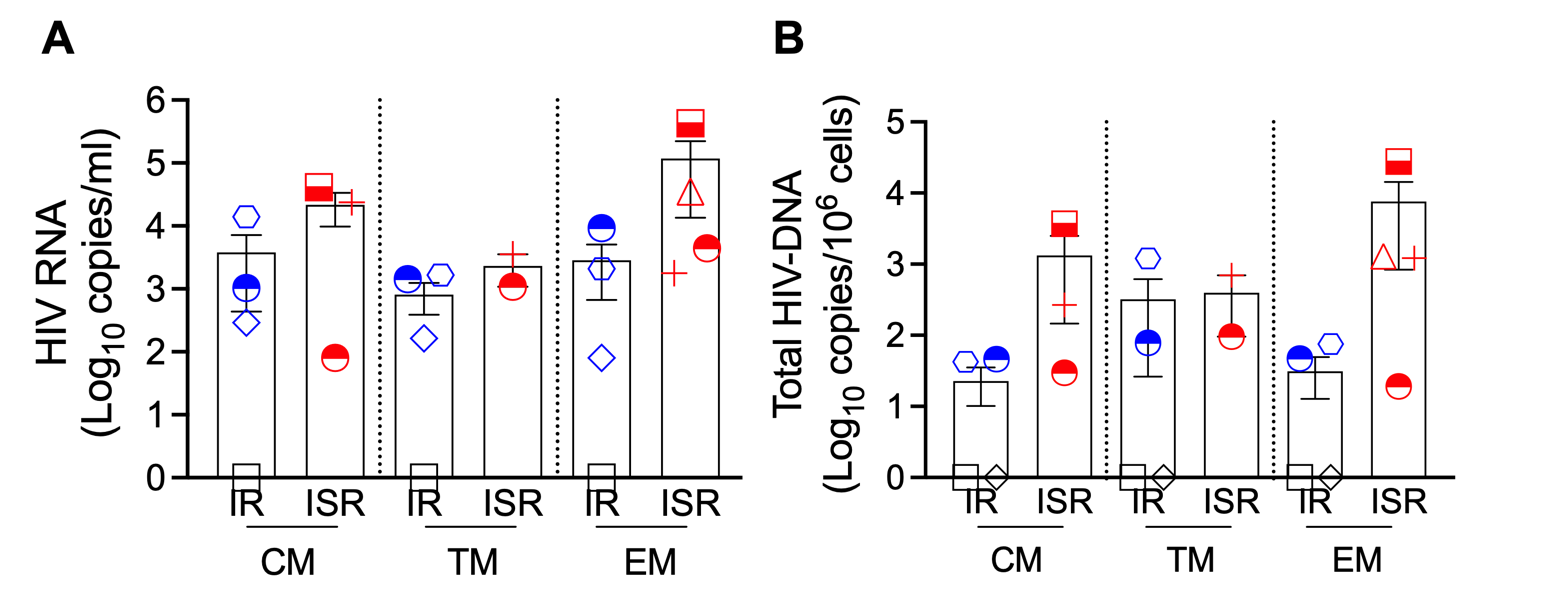

Supplement: S12 Fig — (A) HIV-RNA supernatant, and (B) cell-associated HIV-DNA of sorted CD4 T-cell subsets after 5 days of IL-15+IL7 stimulation from on-ART ISR (red, n = 3) and IR (blue, n = 4) individuals. CD4 T-cell subsets included naïve (N), central memory (CM), transitional memory TM, and effector memory (EM) cells. Black symbols correspond to undetectable values. Averaged data are presented as the mean ± SEM. Mann Whitney u-test was used for statistical analysis. (TIF) [file ppat.1009825.s012.tif]
