# Supplementary material for: Increased homeostatic cytokines and stability of HIV-infected memory CD4 T-cells identify individuals with suboptimal CD4 T-cell recovery on-ART
Source: PLoS Pathog. 2021 Aug 27;17(8):e1009825. doi: 10.1371/journal.ppat.1009825 (PMC8397407; doi:10.1371/journal.ppat.1009825)
Supplement: S1 Table — (DOCX) [file ppat.1009825.s013.docx]

**S1 Table. Demographic and clinical characteristics of the study participants.**

| **Characteristics** | **Total Cohort (n=32)** | **Immunologic Responders (n=13)** | **Immunologic Suboptimal Responders (n=19)** | **P-value^1^** |
| --- | --- | --- | --- | --- |
| ***Sex*** | | | | |
| Female | 3 (9%) | 1 (8%) | 2 (11%) | 1 |
| Male | 29 (91%) | 12 (92%) | 17 (89%) |  |
| ***Age*** | 50 ± 11 | 51 ± 14 | 49 ± 10 | 0.68 |
| ***Ethnicity*** | | | | |
| African/American | 23 (72%) | 9 (69%) | 14 (74%) | 1 |
| White/Caucasian | 5 (16%) | 2 (15%) | 3 (16%) |  |
| Hispanic | 4 (13%) | 2 (15%) | 2 (11%) |  |
| Asian | 0 (0%) | 0 (0%) | 0 (0%) |  |
| Other | 0 (0%) | 0 (0%) | 0 (0%) |  |
| ***CD4 nadir, cells/mm^3 2^*** | 182 ± 145 | 321 ± 109 | 86 ± 67 | <0.0001 |
| ***CD4 baseline, cells/mm^3^*** | 217 ± 179 | 390 ± 140 | 99 ± 76 | <0.0001 |
| ***Pre-ART CD4/CD8 ratio^3^*** | 0.35 ± 0.31 | 0.58 ± 0.35 | 0.18 ± 0.13 | 0.0074 |
| ***On-ART CD4/CD8 ratio^3^*** | 0.83 ± 0.5 | 1.1 ± 0.53 | 0.62 ± 0.36 | 0.02 |
| ***Duration of ART, years*** | 4 ± 2 | 3 ± 2 | 4 ± 3 | 0.29 |
| ***Time from HIV diagnosis to ART initiation, years*** | 4.3 ± 4.1 | 3.7 ± 3.4 | 4.9 ± 4.8 | 0.62 |
| ***Baseline plasma HIV Viral load^4^*** | 4.418 ± 1.319 | 4.254 ± 1.665 | 4.623 ± 0.760 | 0.57 |
| ***ART*** |  |  |  | 0.21 |
| Two NRTIs, and a boosted PI | 23 (72%) | 8 (62%) | 15 (79%) |  |
| Two NRTIs, an INSTI, and a PK enhancer | 0 | 0 | 0 |  |
| Two NRTIs, an INSTI | 1 (3%) | 0 | 1 (5%) |  |
| Two NRTIs and an NNRTI | 8 (25%) | 5 (38%) | 3 (16%) |  |
| ***AIDS defining illness at pre-ART*** | 6 (19%) | 0 | 6 (33%) | 0.03 |
| ***Co-infections*** | 2 (8%) | 1 (8%) | 1 (5%) | 1.00 |
| Continuous variables are reported as mean ± SD and categorical variables are reported as no. (%) | | | | |
| ^1^Groups are compared with a two-sample t-test for continuous variables, Exact Chi-square test for categorical variables and Two-Sample Wilcoxon Rank-Sum Test for non-normally distributed variables.  ^2^Lowest CD4 count registered during the follow-up of the patient.  ^3^ CD4/CD8 ratios were available for 18 participants (8 IRs, and 10 ISRs). | | | | |
| ^4^Baseline Plasma HIV viral load data are Log_10_ transformed and reported as Log_10_ mean + standard deviation.  ART, antiretroviral therapy; NRTIs, nucleoside reverse transcriptase inhibitors; PI, protease inhibitor; INSTI, integrase inhibitor; NNRTI, non-nucleoside reverse transcriptase inhibitors. | | | | |
