# Supplementary material for: Increased homeostatic cytokines and stability of HIV-infected memory CD4 T-cells identify individuals with suboptimal CD4 T-cell recovery on-ART
Source: PLoS Pathog. 2021 Aug 27;17(8):e1009825. doi: 10.1371/journal.ppat.1009825 (PMC8397407; doi:10.1371/journal.ppat.1009825)
Supplement: S2 Table — Data show statistical differences between IR and ISR. SD, standard deviation. (DOCX) [file ppat.1009825.s014.docx]

**S2 Table.** **List of cytokines measured in plasma at pre-ART time point.** Data show statistical differences between IR and ISR. SD, standard deviation.

| Cytokine | p-Value | SD |
| --- | --- | --- |
| **IL-7** | 0.022144522 | 1.414741431 |
| **IL-15** | 0.034965035 | 0.909085209 |
| **IL-4** | 0.073426573 | 0.04489432 |
| **TRAIL** | 0.180652681 | 75.26822915 |
| **VEGF** | 0.180652681 | 1.99659758 |
| **IFN-g** | 0.234265734 | 0.587117023 |
| **IL-18** | 0.234265734 | 195.2670331 |
| **IL-6** | 0.234265734 | 0.232458379 |
| **IFNa2a** | 0.294871795 | 0.501407661 |
| **IL-22** | 0.294871795 | 147.707551 |
| **TNF-a** | 0.365967366 | 0.541003282 |
| **IL-9** | 0.4744429 | 0.084194017 |
| **TGF-b3** | 0.4744429 | -0.15783263 |
| **IL-17a** | 0.51448258 | -0.069644285 |
| **IL-29** | 0.533799534 | 4.551999483 |
| **IFNb** | 0.562290035 | 1.563997087 |
| **TGF-b1** | 0.628205128 | -262.703728 |
| **IL-1a** | 0.699187938 | 5.29E-06 |
| **TGF-b2** | 0.829398637 | -1.35854392 |
| **IL-1b** | 0.909583275 | -4.24E-06 |
| **IL-21** | 0.930478815 | -1.16E-05 |
| **IL-10** | 1 | -0.02683652 |
