# Supplementary material for: Increased homeostatic cytokines and stability of HIV-infected memory CD4 T-cells identify individuals with suboptimal CD4 T-cell recovery on-ART
Source: PLoS Pathog. 2021 Aug 27;17(8):e1009825. doi: 10.1371/journal.ppat.1009825 (PMC8397407; doi:10.1371/journal.ppat.1009825)
Supplement: S3 Table — (DOCX) [file ppat.1009825.s015.docx]

**S3 Table.** **Raw means and 95% confidence intervals for HIV-DNA levels in blood (PBMC) CD4 T-cell subsets (Naïve, CM, TM and EM).**

| **CD4 T-cell subset** | **Pre-ART** | | | |  | **On-ART** | | |
| --- | --- | --- | --- | --- | --- | --- | --- | --- |
|  |  | N | Log_10_ Mean ± SEM | P-value |  | N | Log_10_ Mean ± SEM | P-value |
| Naïve  (total) | IR | 10 | 2.469+0.35 | 0.0021 |  | 13 | 1.977+0.239 | 0.0087 |
|  | ISR | 8 | 4.025+0.137 |  |  | 19 | 2.836+0.126 |  |
|  |  |  |  |  |  |  |  |  |
| Naïve (integrated) | IR | 10 | 1.55+0.298 | 0.0043 |  | 13 | 1.218+0.145 | <0.0001 |
|  | ISR | 8 | 2.811±0.179 |  |  | 18 | 2.199+0.14 |  |
|  |  |  |  |  |  |  |  |  |
| CM (total) | IR | 10 | 3.51+0.269 | 0.0676 |  | 13 | 2.416+0.24 | 0.0003 |
|  | ISR | 8 | 4.352+ 0.141 |  |  | 19 | 3.427+0.097 |  |
|  |  |  |  |  |  |  |  |  |
| CM (integrated) | IR | 10 | 3.029+ 0.242 | 0.0085 |  | 13 | 2.047+0.252 | 0.0002 |
|  | ISR | 8 | 3.979+ 0.0729 |  |  | 19 | 3.205+0.121 |  |
|  |  |  |  |  |  |  |  |  |
| TM  (total) | IR | 10 | 3.676+ 0.274 | 0.161 |  | 13 | 2.534+0.238 | 0.0007 |
|  | ISR | 7 | 4.283+ 0.158 |  |  | 19 | 3.459+0.109 |  |
|  |  |  |  |  |  |  |  |  |
| TM (integrated) | IR | 10 | 3.317+ 0.23 | 0.025 |  | 13 | 2.246+0.189 | 0.0001 |
|  | ISR | 7 | 4.03+ 0.089 |  |  | 19 | 3.26+0.112 |  |
|  |  |  |  |  |  |  |  |  |
| EM (total) | IR | 10 | 3.41+ 0.342 | 0.055 |  | 13 | 2.477+0.275 | 0.0019 |
|  | ISR | 7 | 4.275+ 0.246 |  |  | 19 | 3.423+ 0.133 |  |
|  |  |  |  |  |  |  |  |  |
| EM (integrated) | IR | 10 | 2.996+ 0.264 | 0.0046 |  | 13 | 2.476+ 0.252 | 0.0045 |
|  | ISR | 7 | 4.256+ 0.136 |  |  | 19 | 3.431+ 0.124 |  |
