# Supplementary material for: Increased homeostatic cytokines and stability of HIV-infected memory CD4 T-cells identify individuals with suboptimal CD4 T-cell recovery on-ART
Source: PLoS Pathog. 2021 Aug 27;17(8):e1009825. doi: 10.1371/journal.ppat.1009825 (PMC8397407; doi:10.1371/journal.ppat.1009825)
Supplement: S4 Table — The total of LN biopsies available were 14 total, 7 for immunological responders (IR), and 7 for immunological suboptimal responders (ISR). (DOCX) [file ppat.1009825.s016.docx]

**S4 Table.** **Inclusion and exclusion criteria for participation in the lymph node biopsy on-ART.** The total of LN biopsies available were 14 total, 7 for immunological responders (IR), and 7 for immunological suboptimal responders (ISR).

| INCLUSION CRITERIA | EXCLUSION CRITERIA |
| --- | --- |
| 1) HIV-positive individual aged 18 years or older  2) Able to provide informed consent in English  3) Remaining on continuous ART with virologic suppression for at least 36 months at the time of enrollment  a) ART defined as at least two FDA-approved antiretrovirals  b) Continuous ART defined as no interruptions of antiretrovirals for more than 2 weeks and switches allowed for toxicity  c) Virologic suppression defined as no HIV VL > 1000 copies/mL after initial VL below assay detection limits. Transient viremic blips do not exclude participation if flanked by viral levels below detection limits  4) Willing to undergo LN biopsy sampling  5) For those individuals not receiving care from an Emory University clinical affiliate, documentation of HIV positive status for participant | 1) Significant laboratory abnormalities prior to study visit for LN biopsies, including but not limited to:  a) Hemoglobin ≤ 10 g/dL  b) PTT > 1.5x upper limit of normal or INR > 1.5x upper limit of normal  c) Platelet count <100,000  2) Any known medical condition that, in the judgment of the investigators, increases the risk of local or systemic complications of biopsy procedure, including but not limited to:  a) Ongoing pregnancy  b) History of uncontrolled bleeding diathesis  c) Evidence on clinical examination of ulcerative, suppurative, or proliferative lesions of the skin in the inguinal area, or untreated sexually transmitted disease with inguinal involvement  d) History of Injection Drug Use  3) Continued need for, or use during the 14 days prior to enrollment, of the following medications:  a) Aspirin or more than 4 doses of non-steroidal anti-inflammatory drugs  b) Warfarin, heparin (low-molecular weight or unfractionated), platelet aggregation inhibitors, or fibrinolytic agents  4) Continued need for, or use during the 90 days prior to enrollment, of the following medications:  a) Systemic immunomodulatory agents  b) Supraphysiologic doses of steroids  c) Experimental medications, vaccines, or biologicals |
