# Supplementary material for: Increased homeostatic cytokines and stability of HIV-infected memory CD4 T-cells identify individuals with suboptimal CD4 T-cell recovery on-ART
Source: PLoS Pathog. 2021 Aug 27;17(8):e1009825. doi: 10.1371/journal.ppat.1009825 (PMC8397407; doi:10.1371/journal.ppat.1009825)
Supplement: S5 Table — (DOCX) [file ppat.1009825.s017.docx]

**S5 Table.** **Demographic and clinical characteristics of the study participants with** **lymph node biopsy on-ART available.**

| **Characteristics** | **Total LN (n=14)** | **Immunologic Responders (n=7)** | **Immunologic Suboptimal Responders (n=7)** | **P-value^1^** |
| --- | --- | --- | --- | --- |
| ***Sex*** | | | | |
| Female | 0 (0%) | 0 (0%) | 0 (0%) | 1 |
| Male | 14 (100%) | 7 (100%) | 7 (100%) |  |
| ***Age*** | 42 ± 9 | 45 ± 9 | 39 ± 10 | 0.22 |
| ***Ethnicity*** | | | | |
| African/American | 11 (79%) | 6 (86%) | 5 (71%) | 1 |
| White/Caucasian | 3 (21%) | 1 (14%) | 2 (29%) |  |
| Hispanic | 0 (0%) | 0 (0%) | 0 (0%) |  |
| Asian | 0 (0%) | 0 (0%) | 0 (0%) |  |
| Other | 0 (0%) | 0 (0%) | 0 (0%) |  |
| ***CD4 nadir, cells/mm^3 2^*** | 191 ± 146 | 301 ± 101 | 80 ± 87 | 0.0041 |
| ***CD4 baseline, cells/mm^3^*** | 237 ± 206 | 392 ± 171 | 83 ± 84 | 0.0023 |
| ***Duration of ART, years*** | 4 ± 3 | 3 ± 3 | 5 ± 4 | 0.56 |
| ***ART*** |  |  |  | 1 |
| Two NRTIs, and a boosted PI | 11 (79%) | 5 (71%) | 6 (86%) |  |
| Two NRTIs, an INSTI, and a PK enhancer | 0 | 0 | 0 |  |
| Two NRTIs, an INSTI | 0 | 0 | 0 |  |
| Two NRTIs and an NNRTI | 3 (21%) | 2 (29%) | 1 (14%) |  |
| Continuous variables are reported as mean ± SD and categorical variables are reported as no. (%) | | | | |
| ^1^Groups are compared with a two-sample t-test for continuous variables, Exact Chi-square test for categorical variables and Two-Sample Wilcoxon Rank-Sum Test for non-normally distributed variables.  ^2^Lowest CD4 count registered during the follow-up of the patient. | | | | |
| ART, antiretroviral therapy; NRTIs, nucleoside reverse transcriptase inhibitors; PI, protease inhibitor; INSTI, integrase inhibitor; NNRTI, non-nucleoside reverse transcriptase inhibitors. | | | | |
